# Supplementary material for: Neural surprise in somatosensory Bayesian learning
Source: PLoS Comput Biol. 2021 Feb 2;17(2):e1008068. doi: 10.1371/journal.pcbi.1008068 (PMC7880500; doi:10.1371/journal.pcbi.1008068)
Supplement: S1 Appendix — In this supplementary text we provide the derivations for the presented equations of the compared Bayesian learner models. (PDF) [file pcbi.1008068.s001.pdf]

S1 Appendix. Bayesian learner models. In this supplementary text we provide the derivations for the presented equations of the compared Bayesian learner models.

Dirichlet-Categorical model

Given a sequence of observations $y_1, \ldots, y_t$ the Dirichlet-Categorical model combines the likelihood with the prior to refine the posterior estimates over the latent variable space (equation 4):

$$\begin{aligned} p(s_1, \ldots, s_M | y_1, \ldots, y_t) &\propto p(s_1, \ldots, s_M | \ \alpha_1, \ldots, \alpha_M) \prod_{i=1}^{t} p(y_i | s_1, \ldots, s_M) \\ &= \prod_{j=1}^{M} s_j^{\alpha_j - 1} \prod_{i=1}^{t} \prod_{j=1}^{M} s_j^{\mathbf{1}\{y_i = j\}} \\ &= \prod_{j=1}^{M} s_j^{\alpha_j - 1 + \sum_{i=1}^{t} \mathbf{1}\{y_i = j\}} \end{aligned}$$

The posterior predictive distribution in equation 6 can be obtained by integrating over the space of latent states:

$$\begin{aligned} p(y_t = x | y_1, \ldots, y_{t-1}) &= \int p(y_t = x | s_1, \ldots, s_M) p(s_1, \ldots, s_M | y_1, \ldots, y_{t-1}) d\mathcal{S}_M \\ &= \int s_x \frac{\Gamma(\sum_{j=1}^{M} \alpha_j^t)}{\prod_{j=1}^{M} \Gamma(\alpha_j^t)} \prod_{j=1}^{M} s_j^{\alpha_j^t - 1} d\mathcal{S}_M \\ &= \frac{\Gamma(\sum_{j=1}^{M} \alpha_j^t)}{\prod_{j=1}^{M} \Gamma(\alpha_j^t)} \int \prod_{j=1}^{M} s_j^{\mathbf{1}\{x=j\} + \alpha_j^t - 1} d\mathcal{S}_M \\ &= \frac{\Gamma(\sum_{j=1}^{M} \alpha_j^t)}{\prod_{j=1}^{M} \Gamma(\alpha_j^t)} \frac{\prod_{j=1}^{M} \Gamma(\mathbf{1}\{x = j\} + \alpha_j^t)}{\Gamma(1 + \sum_{j=1}^{M} \alpha_j^t)} \\ &= \frac{\alpha_x^t}{\sum_{j=1}^{M} \alpha_j^t} \end{aligned}$$

The surprise readout functions for the Categorical-Dirichlet model introduced in equations 9 to 11 are:

Predictive Surprise

$$\begin{aligned} PS(y_t) &= -\ln p(y_t | y_1, \ldots, y_{t-1}) \\ &= -\ln \left(\prod_{j=1}^{M} \left(\frac{\alpha_x^t}{\sum_{j=1}^{M} \alpha_j^t} \right)^{\mathbf{1}\{y_t = j\}} \right) \end{aligned}$$

Bayesian Surprise

$$BS(o_t) \coloneqq KL(p(s_{t-1} | y_1, \ldots y_{t-1}) || p(s_t | y_1, \ldots, y_t))$$

The general KL divergence for two Dirichlet distributions P and Q parametrized by $\{\alpha_m\}_{m=1}^{M}$ and $\{\alpha'_m\}_{m=1}^{M}$ is given by

$$
\begin{aligned}
KL(P||Q) =& \mathbb{E}_{p(x)}[\log P(x) - \log Q(x)] \\
=& \mathbb{E}_{p(x)}[\log \Gamma(\sum_m \alpha_m) - \sum_m \log \Gamma(\alpha_m) + \sum_m (\alpha_m - 1) \log x_m \\
& - \log \Gamma(\sum_m \alpha'_m) + \sum_m \log \Gamma(\alpha'_m) - \sum_m (\alpha'_m - 1) \log x_m] \\
=& \log \Gamma(\sum_m \alpha_m) - \sum_m \log \Gamma(\alpha_m) - \log \Gamma(\sum_m \alpha'_m) + \sum_m \log \Gamma(\alpha'_m) \\
& - \sum_m (\alpha_m - \alpha'_m) \left(\psi(\alpha_m) - \psi(\sum_m \alpha_m) \right)
\end{aligned}
$$

where $\psi(.)$ denotes the digamma function.

Confidence-Corrected Surprise

$$CS(o_t) \coloneqq KL(p(s_{t-1}|y_1, \ldots, y_{t-1})||\hat{p}(s_t|y_t))$$

The flat prior can be written as $Dir(\alpha_1, \ldots, \alpha_m)$ where $\alpha_m = 1$ for all $m = 1, \ldots, M$. The naive observer posterior simply updates the flat prior based on only the most recent observation y_t. Hence, we have that $\hat{p}(s_t|y_t) = Dir(\alpha'_1, \ldots, \alpha'_m)$ with $\hat{\alpha}_m = 1 + \mathbf{1}_{y_t=m}$. Hence at a given point in time t, we have:

$$
\begin{aligned}
KL(p(s_{t-1}|y_1, \ldots, y_{t-1})||\hat{p}(s_t|y_t)) =& \log \Gamma(\sum_m \alpha_m^{t-1}) - \sum_m \log \Gamma(\alpha_m^{t-1}) - \log \Gamma(\sum_m \alpha_m^t) \\
& + \sum_m \log \Gamma(\alpha_m^t) \\
& - \sum_m (\alpha_m^{t-1} - \alpha_m^t) \left(\psi(\alpha_m^{t-1}) - \psi(\sum_m \alpha_m^{t-1}) \right)
\end{aligned}
$$

Hidden Markov Model

For the use of parameter inference via the expectation-maximisation algorithm and in order to derive the factorisation of the joint likelihood $p(o_{1:t}, s_{1:t})$, we will make sure of the following derivations:

$$
\begin{aligned}
p(s_t|o_1, \ldots, o_T) &= \frac{p(o_1, \ldots o_t|s_t)p(o_{t+1}, \ldots, o_T|s_t)p(s_t)}{p(o_1, \ldots, o_T)} \\
&= \frac{p(o_1, \ldots o_t, s_t)p(o_{t+1}, \ldots, o_T|s_t)}{p(o_1, \ldots, o_T)} \\
&= \frac{\alpha(s_t)\beta(s_t)}{p(o_1, \ldots, o_T)}
\end{aligned}
$$

where for the final line we have redefined the backward and forward probabilities as

$$
\begin{aligned}
\alpha(s_t) &\coloneqq p(o_1, \ldots o_t, s_t) \\
\beta(s_t) &\coloneqq p(o_{t+1}, \ldots, o_T|s_t) \ .
\end{aligned}
$$

In the following, we derive the forward and backward equations which may be used in conjunction with a Dynamic Programming paradigm such as the Baum-Welch algorithm in order to perform the Expectation-Maximisation inference procedure.

$$\begin{aligned}
\alpha(s_t) &= p(o_1, \ldots, o_t, s_t) = p(o_t|s_t)p(o_1, \ldots, o_{t-1}, s_t) \\
&= p(o_t|s_t) \sum_{s_{t-1}} \alpha(s_{t-1}) p(s_t|s_{t-1}) \\
\alpha(s_1) &= p(s_1)p(o_1|s_1) = \prod_{k=1}^{K} \{\pi_k p(o_1|s_{1k})\}^{s_{1k}}
\end{aligned}$$

$$\begin{aligned}
\beta(s_t) &= p(o_{t+1}, \ldots, o_T|s_t) = \sum_{s_{t+1}} p(o_{t+1}, \ldots, o_T, s_{t+1}|s_t) \\
&= \sum_{s_{t+1}} p(o_{t+2}, \ldots, o_T|s_{t+1}) p(o_{t+1}|s_{t+1}) p(s_{t+1}|s_t) \\
&= \sum_{s_{t+1}} \beta(s_{t+1}) p(o_{t+1}|s_{t+1}) p(s_{t+1}|s_t) \\
\beta(s_T) &= 1
\end{aligned}$$

Both expressions for α and β involve a backward and forward recursion. Given a sequence of observations, these can easily be computed in a sequential fashion. The final quantity of interest for the EM algorithm are the smoothed transition probabilities:

$$p(s_{t-1}, s_t|o_1, \ldots o_T) = \frac{\alpha(s_{t-1})p(o_t|s_t)p(s_t|s_{t-1})\beta(s_t)}{p(o_1, \ldots, o_T)}$$

In order to infer the parameters we now alternate between an expectation and a maximisation step:

$$p(o_{1:t}, z_{1:t}) = \prod_{t=1}^{T} p(o_t|s_t) p(s_1) \prod_{t=2}^{T} p(s_t|s_{t-1})$$

1. **Expectation**:

$$\begin{aligned}
\mathbb{E}[\ln p(o_{1:t}, s_{1:t})] =& \sum_{t=1}^{T}\sum_{k=1}^{K} \mathbb{E}[s_{tk}] \ln B_{jk} + \sum_{k=1}^{K} \mathbb{E}[s_{1k}] \ln \pi_k \\
&+ \sum_{t=2}^{T}\sum_{j=1}^{K}\sum_{k=1}^{K} \mathbb{E}[s_{t-1j} s_{tk}] \ln A_{jk} \\
=& \sum_{t=1}^{T}\sum_{k=1}^{K} \frac{\alpha(s_{tk})\beta(s_{tk})}{p(o_1, \ldots, o_T)} \ln B_{jk} + \sum_{k=1}^{K} \frac{\alpha(s_{1k})\beta(s_{1k})}{p(o_1, \ldots, o_T)} \ln \pi_k \\
&+ \sum_{t=2}^{T}\sum_{j=1}^{K}\sum_{k=1}^{K} \frac{\alpha(s_{t-1j}) \prod_{m=1}^{M} B_{mk}^{\mathbf{1}_{o_t=m}} A_{jk} \beta(s_{tk})}{p(o_1, \ldots, o_T)} \ln A_{jk}
\end{aligned}$$

2. **Maximization**: The Lagrangian with the necessary constraints is determined (i.e. row stochasticity and proper distributions) and the derivatives with respect

to the set of parameters ($\theta = \{A_{jk}, B_{mk}, \pi_k\}$) is computed.

$$\mathcal{L}(\theta) \coloneqq \mathbb{E}[\ln p(o_{1:t}, z_{1:t})] + \lambda(\sum_k \pi_k - 1) + \sum_{m=1}^{M} \lambda_m^B(\sum_k B_{mk} - 1)$$
$$+ \sum_{k=1}^{K} \lambda_k^A(\sum_j A_{kj} - 1)$$

The filtering equation can then be written as

$$p(s_t|o_1, \ldots, o_t) = \frac{p(o_1, \ldots, o_t, s_t)}{p(o_1, \ldots, o_t)} = \frac{\alpha(s_t)}{\sum_{s_t} \alpha(s_t)}$$

Finally, the evaluation is then easily obtained by marginalising over the hidden state:

$$p(o_1, \ldots, o_t) = \sum_{s_t} \alpha(s_t)$$

For timestep t the HMM was fit for a stimulus sequence $o_1, ..., o_t$ which gives a set of parameter estimates, $\hat{\pi}_t, \hat{A}_t, \hat{B}_t$ and the filtering posterior $\hat{\gamma}_t(s_t) = p(s_t|o_1, ..., o_t)$. The predictive surprise as formulated in equation 12 is derived in the following way:

$$\begin{aligned} PS(o_{t+1}) &\coloneqq -\ln p(o_{t+1}|s_{t+1}) = -\ln p(o_{t+1}|o_1, \ldots, o_t) \\ &= -\ln(p(o_{t+1}|s_{t+1})p(s_{t+1}|s_t)p(s_t|o_1, ..., o_t)) \\ &\approx -\ln(\hat{B}_t^T \hat{A}_t^T \hat{\gamma}_t(s_t)) \end{aligned}$$

The Bayesian surprise from equation 13, on the other hand, derives for the HMM as follows:

$$\begin{aligned} BS(o_{t+1}) &\coloneqq KL(p(s_t|o_t, \ldots o_1)||p(s_{t+1}|o_{t+1}, \ldots, o_1)) \\ &= \sum_k^K p(s_t|o_t, \ldots o_1) \ln \frac{p(s_t|o_t, \ldots o_1)}{p(s_{t+1}|o_{t+1}, \ldots, o_1)} \\ &\approx \sum_k^K \hat{\gamma}_t(s_t = k) \ln \frac{\hat{\gamma}_t(s_t = k)}{\hat{\gamma}_{t+1}(s_{t+1} = k)} \end{aligned}$$

Finally, confidence corrected surprise from equation 14 may be expressed as a linear combination of predictive surprise, Bayesian surprise, a model commitment term (negative entropy) $C(p(s_t))$, and a data-dependent constant scaling the state space $O(t)$.

$$\begin{aligned} C(p(s_{t+1}|o_1, ..., o_{t+1})) &= -H(p(s_{t+1}|o_1, ..., o_{t+1})) \\ &= \sum_{k=1}^K p(s_{t+1} = k|o_1, ..., o_{t+1}) \ln(p(s_{t+1} = k|o_1, ..., o_{t+1})) \\ &\approx \sum_{k=1}^K \hat{\gamma}_{t+1}(s_{t+1} = k) \ln \hat{\gamma}_{t+1}(s_{t+1} = k) \end{aligned}$$

$$O(t+1) = \sum_{k=1}^K p(o_{t+1}|s_{t+1}) \approx \sum_{k=1}^K \hat{B}_t^{o_{t+1},k}$$

$$CS(o_t) = BS(o_t) + PS(o_t) + C(p(s_t)) + \ln O(t)$$

All inference types shared the same state space $s \in \mathcal{S} = \{0, 1\}$. Due to the transformation of the observation sequence the observation space differed between models:

Stimulus probability model: $y_t = o_t$ for $t = 1, ..., T$ with $\mathcal{O}_{SP} = \{0, 1\}$

Alternation probability model: $y_t = d_t$ for $t = 2, ..., T$ with $\mathcal{O}_{AP} = \{0, 1\}$ and $d_t = 1_{o_t \neq o_{t-1}}$

Transition probability model 1st Order: $y_t = e_t^i$ for $t = 2, ..., T$ with $\mathcal{O}_{TP1} = \{0, 1, 2, 3\}$ as e_t^i belongs to the set containing each possible transition from $o_{t-1} = i$.

Transition probability model 2nd Order: $y_t = e_t^j$ for $t = 3, ..., T$ with $\mathcal{O}_{TP2} = \{0, 1, 2, 3, 4, 5, 6, 7\}$ as e_t^j belongs to the set containing each possible transition from $o_{t-2} = j$.
