# Supplementary material for: Neural surprise in somatosensory Bayesian learning
Source: PLoS Comput Biol. 2021 Feb 2;17(2):e1008068. doi: 10.1371/journal.pcbi.1008068 (PMC7880500; doi:10.1371/journal.pcbi.1008068)
Supplement: S2 Appendix — In this supplementary text we present the algorithm used to approximate log model evidence for subsequent Bayesian model comparison. (PDF) [file pcbi.1008068.s002.pdf]

S2 Appendix. Variational inference algorithm. In this supplementary text we present the algorithm used to approximate log model evidence for subsequent Bayesian model comparison.

A free-form variational inference algorithm for general linear models with spherical error covariance matrix.

We consider variational inference for probabilistic models of the form

$$p(y, \beta, \lambda) = p(y|\beta, \lambda)p(\beta)p(\lambda),$$

where

$$p(y|\beta, \lambda) := N(y; X\beta, \lambda^{-1}I_n), \quad p(\beta) = N(\beta; 0_p, \alpha^{-1}I_p), \text{ and } p(\lambda) = G(\lambda; \beta_\lambda, \gamma_\lambda).$$

Here, $y \in \mathbb{R}^n$ denotes the observed random variable modeling data, $\beta \in \mathbb{R}^p, \lambda > 0$ denote unobserved random variables modeling regression weights and observation noise precisions, respectively, and $X \in \mathbb{R}^{n\times p}$ denotes a design matrix. The parameter-conditional distribution of y is specified in terms of a multivariate Gaussian density with expectation parameter $X\beta \in \mathbb{R}^n$ and a spherical covariance matrix parameter $\lambda^{-1}I_n$. The marginal (or prior) distribution of β is specified in terms of a multivariate Gaussian density with zero expectation parameter $0_p \in \mathbb{R}^p$ and covariance matrix parameter $\alpha^{-1}I_p$, where $\alpha > 0$ denotes a precision parameter. Finally, the distribution of λ is specified in terms of a Gamma density in its shape and scale parameterization, where $\beta_\lambda, \gamma_\lambda > 0$ denote the shape and scale parameters, respectively.

Model estimation

Application of the free-form variational inference theorem yields an algorithm that, upon convergence, furnishes an approximation to the data-conditional (posterior) parameter distribution of the form

$$q(\beta)q(\lambda) \approx p(\beta, \lambda|y).$$

Here, the variational distributions take the form

$$q(\beta) = N(\beta; m_\beta, S_\beta) \text{ and } q(\lambda) = G(\lambda; b_\lambda, c_\lambda),$$

where $m_\beta \in \mathbb{R}^p$ and $S_\beta \in \mathbb{R}^{p\times p}$ denote the converged variational expectation and covariance parameters, respectively, while $b_\lambda, c_\lambda > 0$ denote the converged variational shape and scale parameters. Finally, the algorithm furnishes, upon convergence, the variational free energy lower bound

$$F\left(q(\beta)q(\lambda)\right) \leq \ln \iint p(y, \beta, \lambda)\, d\lambda\, d\beta = \ln p(y)$$

to the log marginal likelihood, also known as log model evidence.
The algorithm takes the following form
Initialization

0. Set
$$q^{(0)}(\beta) := N\left(\beta; m_\beta^{(0)}, S_\beta^{(0)}\right) \text{ and } q^{(0)}(\lambda) := G\left(\lambda; b_\lambda^{(0)}, c_\lambda^{(0)}\right)$$
with variational parameters
$$m_\beta^{(0)} := 0_p, \quad S_\beta^{(0)} := \alpha^{-1}I_p$$

and

$$b_\lambda^{(0)} := \beta_\lambda, \quad c_\lambda^{(0)} := \frac{n}{2} + \gamma_\lambda,$$

respectively. Define a convergence criterion $\delta > 0$ and a maximum number of iterations n_i.

Iterations

For $i = 1, ..., n_i$ or until convergence is reached

1. <u>$q(\beta)$ update</u>

   Set

   $$q^{(i)}(\beta) := N\left(\beta; m_\beta^{(i)}, S_\beta^{(i)}\right)$$

   where

   $$S_\beta^{(i)} := \left(b_\lambda^{(i-1)} c_\lambda^{(i-1)} X^T X + \alpha I_p\right)^{-1}$$

   and

   $$m_\beta^{(i)} := b_\lambda^{(i-1)} c_\lambda^{(i-1)} S_\beta^{(i)} X^T y$$

2. <u>$q(\lambda)$ update</u>

   Set

   $$q^{(i)}(\lambda) := G\left(\lambda; b_\lambda^{(i)}, c_\lambda^{(i)}\right)$$

   where

   $$b_\lambda^{(i)} := \left(\frac{1}{2}\left(\mathrm{tr}\left(S_\beta^{(i)} X^T X\right) + \left(y - X m_\beta^{(i)}\right)^T \left(y - X m_\beta^{(i)}\right)\right) + \frac{1}{\beta_\lambda}\right)^{-1}$$

   and

   $$c_\lambda^{(i)} := \frac{n}{2} + \gamma_\lambda.$$

   Note that $c_\lambda^{(i)}$ stays constant throughout.

3. <u>$F\left(q(\beta)q(\lambda)\right)$ update</u>

   Set

   $$F^{(i)} := F\left(q^{(i)}(\beta) q^{(i)}(\lambda)\right),$$

   where

   $$F\left(q^{(i)}(\beta) q^{(i)}(\lambda)\right) := L_a^{(i)} - \mathrm{KL}\left(q^{(i)}(\beta) || p(\beta)\right) - \mathrm{KL}\left(q^{(i)}(\lambda) || p(\lambda)\right)$$

   where with the digamma function ψ, $L_a^{(i)}$ denotes the average likelihood term

   $$L_a^{(i)} := -\frac{n}{2}\ln 2\pi - \frac{1}{2} b_\lambda^{(i)} c_\lambda^{(i)} \left(y - X m_\beta^{(i)}\right)^T \left(y - X m_\beta^{(i)}\right) - \frac{1}{2} b_\lambda^{(i)} c_\lambda^{(i)} \mathrm{tr}\left(S_\beta^{(i)} X^T X\right) + \frac{n}{2}\psi\left(c_\lambda^{(i)}\right) + \ln b_\lambda^{(i)}$$

   and $\mathrm{KL}(q(x)||p(x))$ denotes the KL-divergence between the densities $q(x)$ and $p(x)$.

4. <u>Convergence assessment</u>

   If $i > 1$, evaluate

   $$\delta_F = F^{(i)} - F^{(i-1)}.$$

   Then,

   - if $\delta_F < 0$, i.e., the variational free energy has decreased, issue a warning and end the algorithm,
   - if $0 < \delta_F < \delta$, i.e., the variational free energy has increased less than δ, end the algorithm and declare convergence,
   - else go to 1.

Prior variational distributions

In order to select the probabilistic model of interest that minimizes Type II errors under the constraint of minimizing Type I errors the following test procedure was implemented. Data was simulated with low signal-to-noise levels (true, but unknown, $\lambda = 0.001$ and $\beta = [1; 1]$) and underwent z-score normalization, after which model retrieval was evaluated for a range of values for the precision parameter α. For data generated by the null model, a range of values of α was determined for which false positives were highly unlikely (exceedence probability $\phi = 1$ in favour of the null model in every one of the 100 iterations). Next, for data generated by the non-null models, the value of α was selected which lied within the previously established range and for which the difference in log model evidence between null and non-null models was maximized. This procedure yielded the following prior distributions which were used in all described evaluations.

$$q^{(0)}(\beta) := N(\beta; 0_p, 0.001 I_p) \text{ and } q^{(0)}(\lambda) := G(\lambda; 10, 0.1)$$
