# Supplementary figures and images for: Neural surprise in somatosensory Bayesian learning
Source: PLoS Comput Biol. 2021 Feb 2;17(2):e1008068. doi: 10.1371/journal.pcbi.1008068 (PMC7880500; doi:10.1371/journal.pcbi.1008068)

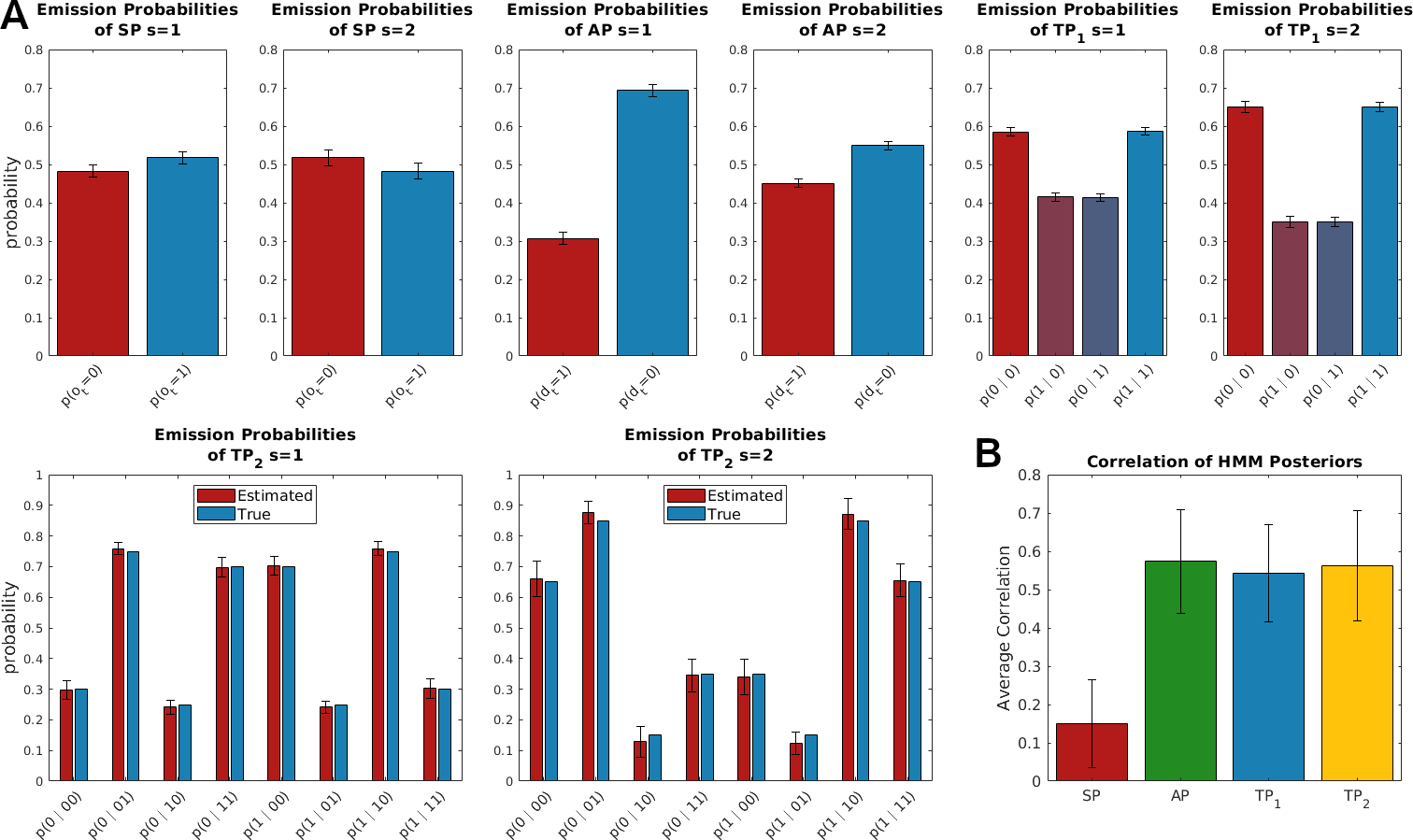

Supplement: S1 Fig — (A) The average emission probabilities of the stimulus probability (SP), alternation probability (AP), and transition probability (TP) hidden Markov model (HMM) for both states (s) at the final timestep of each sequence. For TP2, a comparison is provided of the emission probabilities used for data generation and the average, normalized emission probabilities estimated by the HMM. Error bars represent the standard error of the mean. (B) Correlating the true regimes and filtering posterior over time confirms that AP and TP inference allow for the tracking of the fast and slow-switching regimes, while SP inference does not capture the necessary dependencies due to the regimes being balanced in terms of stimulus probabilities. (TIF) [file pcbi.1008068.s003.tif]

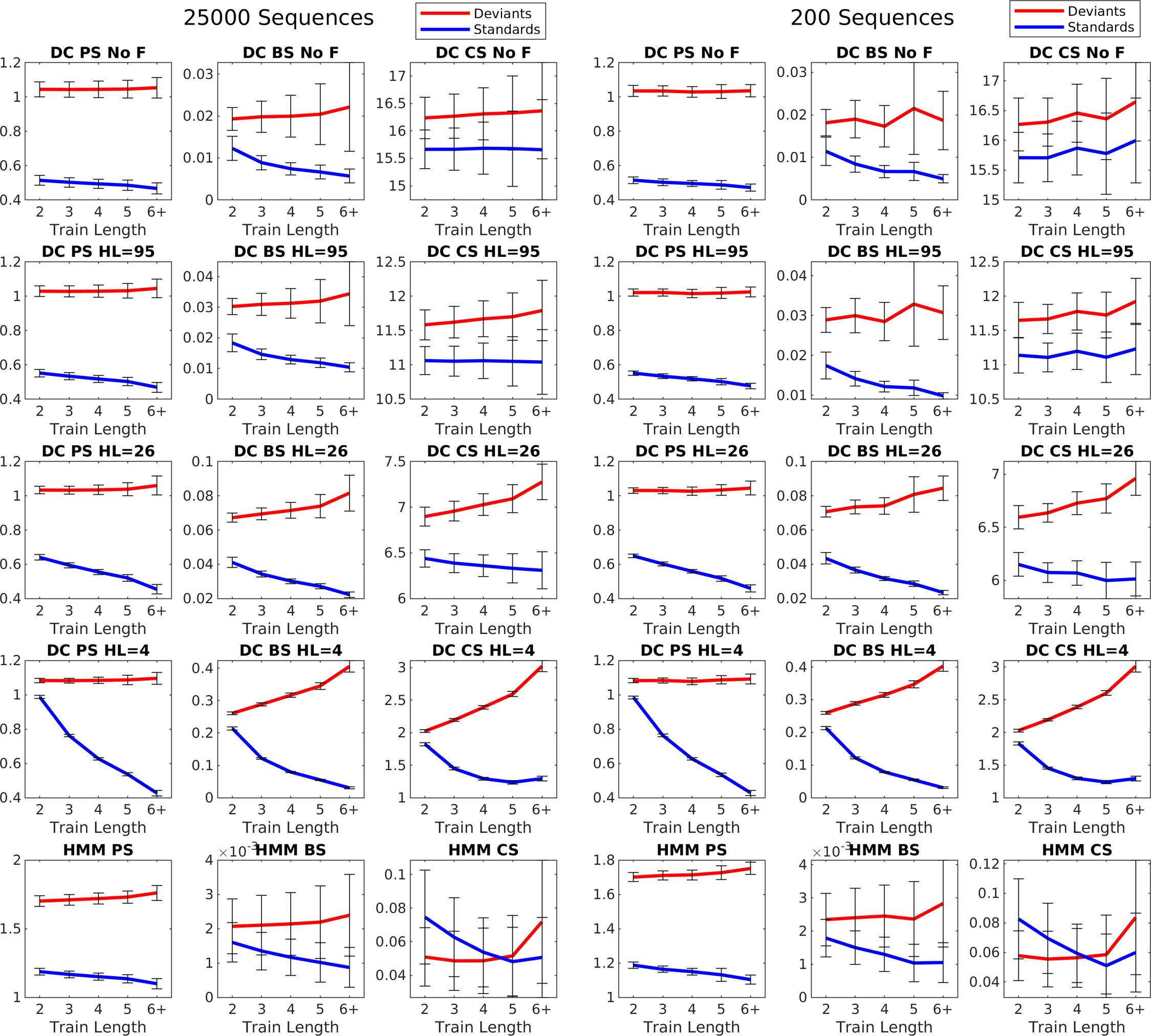

Supplement: S2 Fig — Averaged surprise readouts using either the (left) 25000 total sequences or (right) 200 sequences administered to the participants elicited for standard and deviant stimuli following a certain amount of repeating stimuli (train length). The model-derived predictions are relatively well-preserved in the smaller data-set. Only first-order transition probability models are plotted. Error bars indicate standard deviations. The used stimulus half-lives of 95 and 26 are representative of the winning models in the single-trial EEG analysis. DC: Dirichlet-Categorical model; HMM: Hidden Markov Model; PS: Predictive surprise; BS: Bayesian surprise; CS: Confidence-corrected surprise; No F: model without forgetting (i.e. perfect integration); HL: stimulus half-life. (TIF) [file pcbi.1008068.s004.tif]

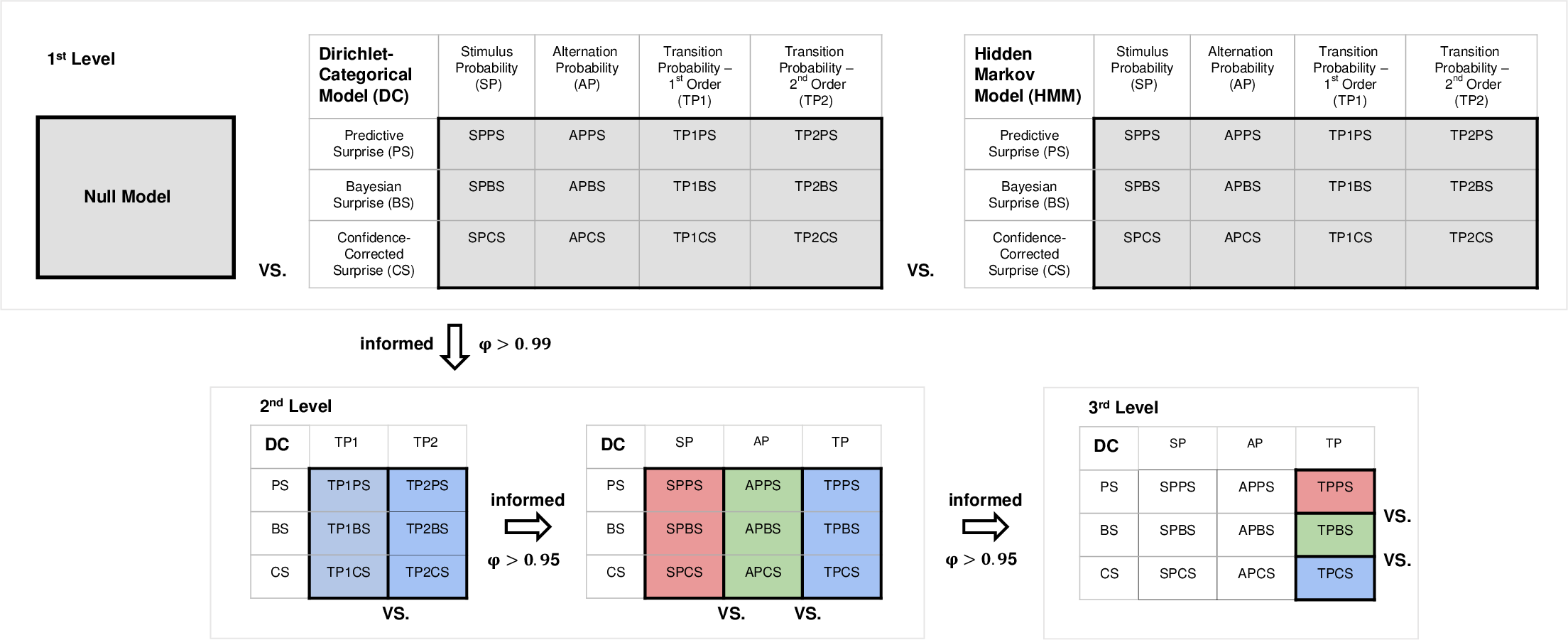

Supplement: S3 Fig — First level (depicted in the top row): The 12 DC models and the 12 HMM models were grouped into their corresponding model class family and compared via BMS against each other and an offset Null-Model. Second level (lower row, left rectangle): Within the DC model class, the two transition probability models TP1 and TP2 were grouped into families and the winner of the BMS was used for the comparison against the other two inference type models (Stimulus Probability (SP) and Alternation Probability (AP)). Third Level (lower row, middle rectangle): The surprise readouts of the DC TP1 model were subjected to BMS and the resulting exceedance probabilities are reported in the main results. Thresholding of the model class families and inference types was again applied at successive levels leading to data reduction. (TIF) [file pcbi.1008068.s005.tif]

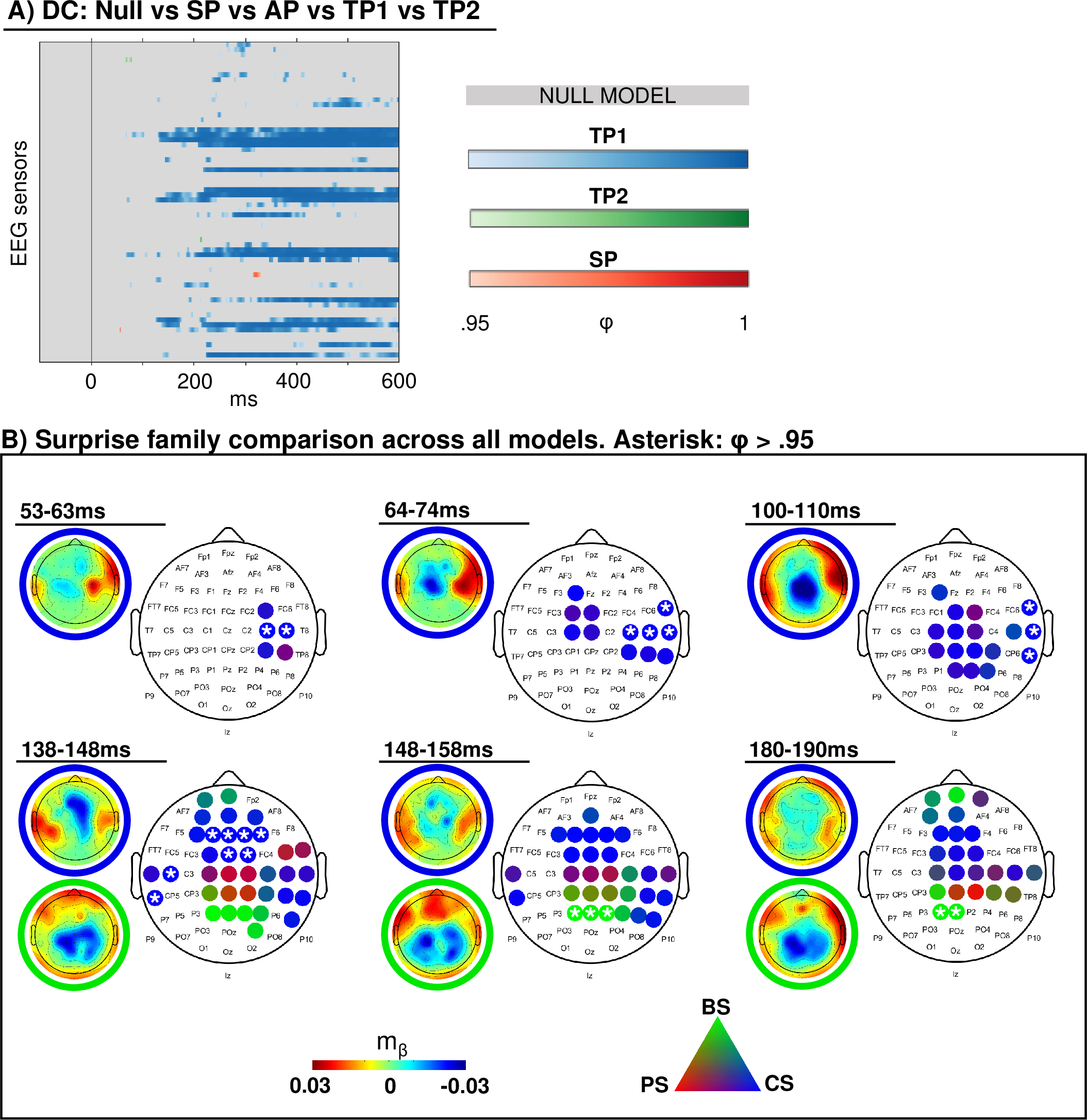

Supplement: S4 Fig — Exceedance probabilities (φ) resulting from the RFX family model comparison by investigating the full model space in each comparison. A) Family comparison of the first order transition probability (TP1), second order transition probability (TP2), alternation probability (AP; no above-threshold results with φ > 0.95) and stimulus probability (SP) models; thresholded at φ > 0.95. B) Unthresholded family comparison of surprise models. Large discrete topographies show the electrode clusters of predictive surprise (PS) in red, Bayesian surprise (BS) in green and confidence-corrected surprise (CS) in blue. White asterisks indicate φ > 0.95. Small continuous topographies display the converged variational expectation parameter (mβ). (TIF) [file pcbi.1008068.s006.tif]

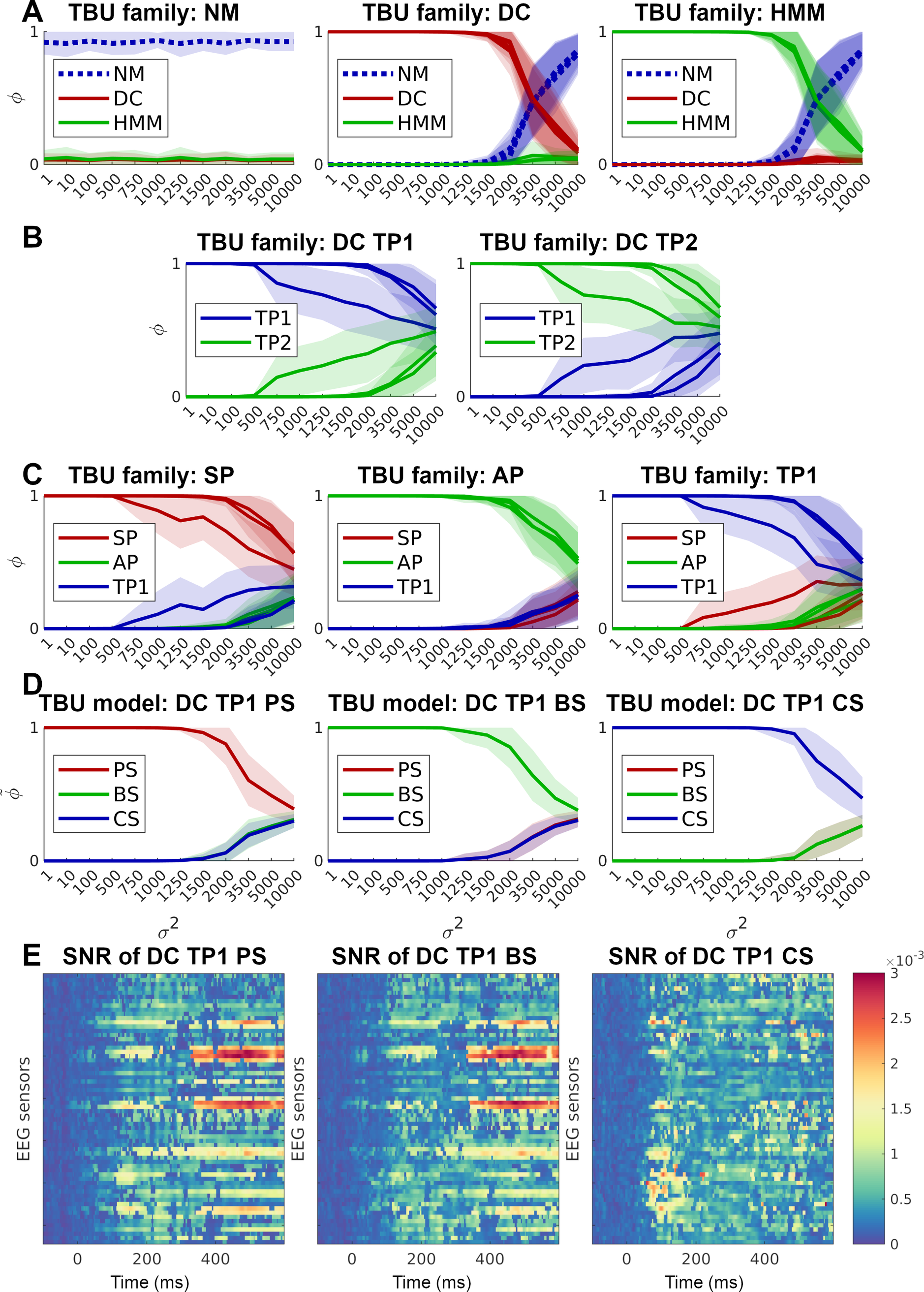

Supplement: S5 Fig — A model recovery study was performed using simulated data. Subplots (A-D) show the average exceedance probabilities (shading represents standard deviations) of 100 random-effects Bayesian model selection analyses under different signal-to-noise ratios. This was performed for (A) Null Model vs DC Model vs HMM families, (B) DC TP1 vs TP2 families, (C) DC SP vs AP vs TP1 families, and (D) DC TP1 PS, BS, and CS models. Noteworthy is that the instances of reduced differentiability for (B) and (C) occurred only when the true, but unknown model was confidence-corrected surprise. (E) An estimate of the signal-to-noise of the experimental single-trial EEG analyses by inspecting the ratio of the expected posterior estimates of the model fitting procedure for β2 and λ−1. (TIF) [file pcbi.1008068.s007.tif]

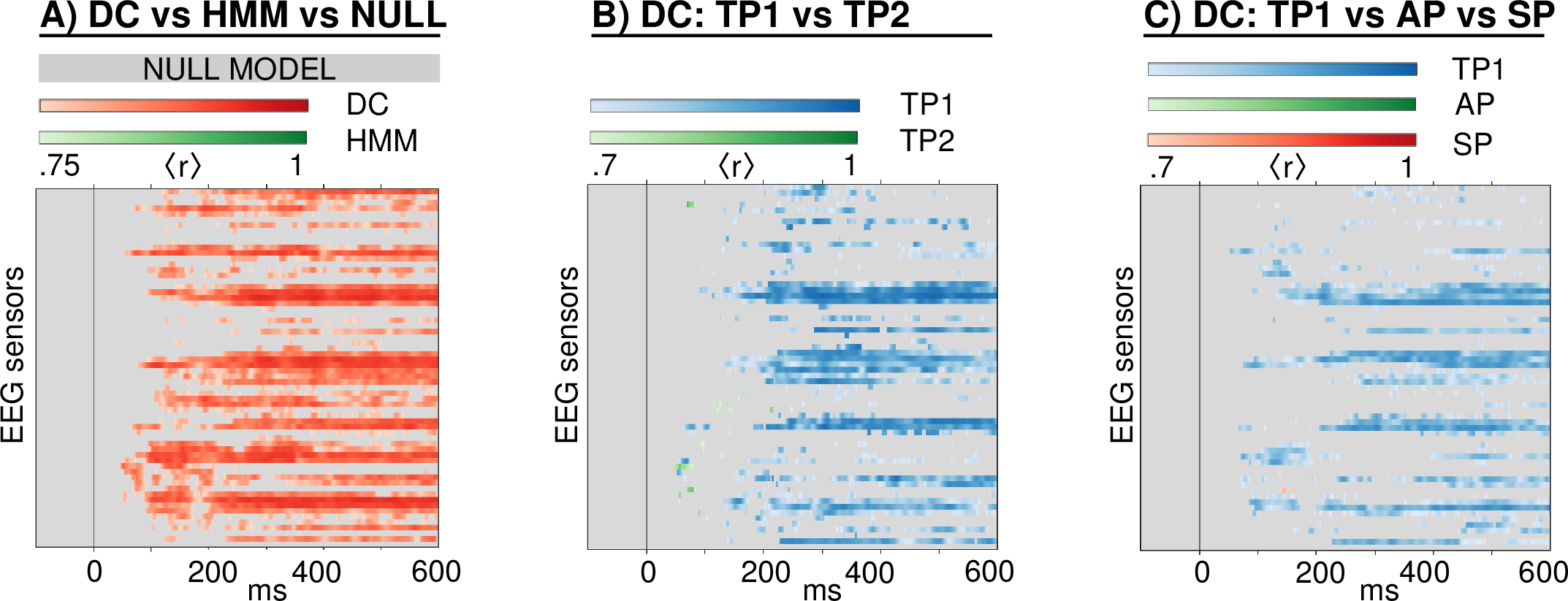

Supplement: S6 Fig — Expected posterior probabilities (〈r〉) resulting from family model comparisons. A) Dirichlet-Categorical (DC) model, Hidden Markov Model (HMM) and Null model family comparison, thresholded at 〈r〉 > 0.75. B) Family comparison within the winning DC family, thresholded at 〈r〉 > 0.7: first and second order transition probability models (TP1, TP2). C) Family comparison within the winning DC family, thresholded at 〈r〉 > 0.7: first order transition probability (TP1), alternation probability (AP) and stimulus probability (SP) models. (TIF) [file pcbi.1008068.s008.tif]

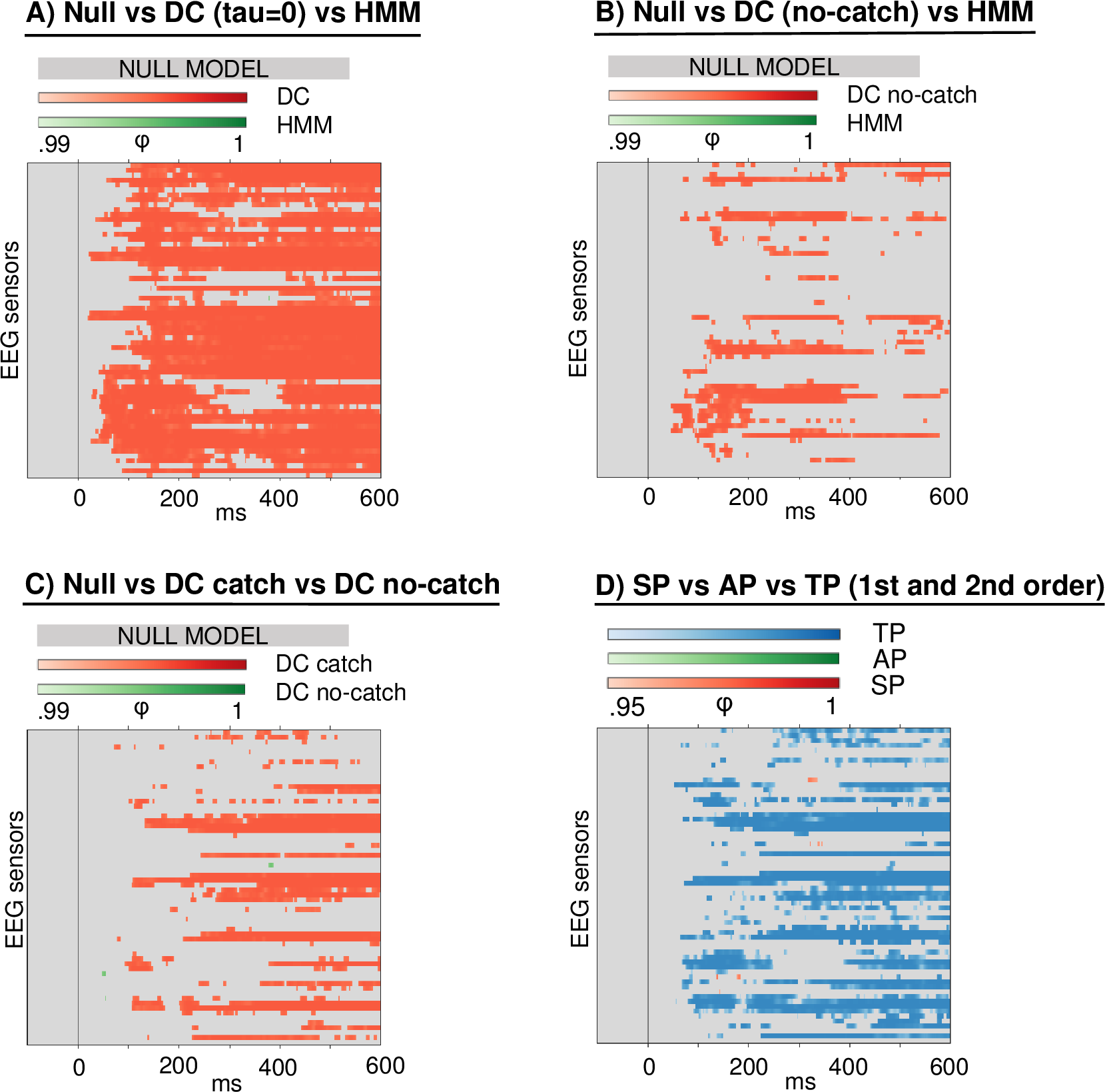

Supplement: S7 Fig — (A) Comparison of the model families: Null model, Dirichlet-Categorical model (DC) with tau = 0 (i.e. no forgetting and no penalization) and Hidden Markov Model (HMM). (B) Comparison of the model families: Null model, DC without modelling the catch trials and HMM. (C) Comparison of the model families: Null model, DC with and DC without modelling the catch trials. (D) Comparison of the model families within the DC model: Stimulus probability model (SP), alternation probability model (AP) and transition probability model family (TP) subsuming first and second order TP models in one family. Exceedance probabilities (φ) are plotted for all comparisons. (TIF) [file pcbi.1008068.s009.tif]
