# Supplementary material for: Neural surprise in somatosensory Bayesian learning
Source: PLoS Comput Biol. 2021 Feb 2;17(2):e1008068. doi: 10.1371/journal.pcbi.1008068 (PMC7880500; doi:10.1371/journal.pcbi.1008068)
Supplement: Attachment — Submitted filename: ResponseToReviewers_PCOMPBIOL-D-20-01012.pdf [file pcbi.1008068.s010.pdf]

Prof. Dr. Samuel Gershman | Dr. Philipp Schwartenbeck

Deputy Editor | Guest Editor

PLoS Computational Biology | PLoS Computational Biology

Revised manuscript: PCOMPBIOL-D-20-01012, “Neural surprise in somatosensory Bayesian learning”

Dear Prof. Dr. Samuel Gershman, dear Dr. Philipp Schwartenbeck,

As encouraged by your letter of July 22, please find attached the revision of our manuscript (PCOMPBIOL-D-20-01012).

We were delighted by the generally positive assessment of our study by the three reviewers and their very clear and helpful comments. We are grateful for the extensive review and insightful suggestions on improving clarity and argumentative strength of the manuscript which undoubtedly resulted from a thorough understanding of the involved methodology. We believe implementing the reviewers’ comments has significantly improved the manuscript.

In brief, considerable additions concern a simulation model recovery study to validate that the models are recoverable in relevant signal-to-noise scenarios using our sequence data and model comparison scheme. Further random-effects model comparison analyses were performed as control analyses to address concerns about our choice of hierarchical ordering of model comparisons and choice of reported statistics. Moreover, we increased the focus on model interpretation and their relation to the conventional ERP analysis results. Finally, we have revised the text considerably to address smaller but very important requests on details of phrasing, spelling mistakes and errors of presentation. Please see our response to the reviews for details of each of the applied changes.

We thank you and the reviewers for your consideration and helpful comments, and hope that you will find our study to be suitable for publication in *PLoS Computational Biology.*

We look forward to your reply.

Sincerely,

Sam Gijsen, Miro Grundei, Robert T. Lange, Dirk Ostwald, Felix Blankenburg

REVIEWER #1

Points for clarification / elaboration

- the Dirichlet-Categorical model, which does not explicitly feature a representation of multiple hidden states and their switches (i.e. is non-hierarchical), was better able to explain the neural data than a Hidden Markov Model (that more accurately corresponds to the true task generative model). The authors comment that perhaps this is evidence that the brain employs simpler (non-hierarchical) perceptual learning models for low level statistical regularity tracking, in the absence of explicit attention to regime switching. I wonder if the authors could comment on how their interpretation interfaces with recent accounts that suggest that even in low-level learning phenomena the brain posits associations between latent causes and observable outcomes (Gershman, Norman and Niv, 2015m Curr. Op. Behav. Sciences).

The authors thank the reviewer for pointing us to the paper of Gershman, Norman and Niv where the authors argue for state representations in reinforcement learning to underlie computations involved in low-level mechanisms such as classical conditioning. The view that state discovery processes play a role for low-level learning in the context of reinforcement learning and corresponding processing of reward based stimuli is not necessarily incompatible with our claim that volatility within a stream of value-free sensory input might be accounted for without the necessity of explicit state representation. A simple forgetting approach might be an effective way to deal with the inputs in the context of our experimental setup and we would not argue against low level hidden state representation in general. We have now included the suggested paper in our manuscript and briefly discuss it in light of our results. Please see the response to the comment below for the suggested change.

- I was surprised that the DC model class remained superior to the HMM class even under conditions of perfect integration (no leak), as the leak parameter is precisely what equips the DC model with an ability to be flexible to changes in task statistics. Is it possible that this result (and the superiority of the DC vs HMM in general) is simply due to the emission probabilities associated with the two hidden states being too similar. If so, this limits how generalizable these findings are to task environments with more noticeable transitions between latent states.

We indeed find superiority of the DC with perfect integration (tau=0) over the HMM and have included a figure of this result in a new supplementary figure collection of control random effects analyses (S7 Fig. A).

Although we primarily interpret this as additional evidence of the apparent insensitivity of the brain to a posterior distribution over latent states as prescribed by the HMM, we acknowledge that the degree of dissimilarity between the hidden states may affect the findings. Despite the HMM retrieving the true, but unknown emission probabilities of the two hidden states on average, they may not be sufficiently distinct for human participants in the current experimental setting. In order to include this possibility, we have adjusted the discussion as follows:

Changes in the revised manuscript (l.707 and l.713) are indicated by a bold fontweight:

In addition to average-based ERP analyses, single-trial brain potentials in response to sequential input can provide a unique window into the mechanisms underlying probabilistic inference in the brain. Here, we investigated the learning of statistical regularities using different Bayesian learner models with single-trial surprise regressors. Partitioning the model space allowed us to infer on distinguishing features between the model families using Bayesian model selection (BMS). The first comparison concerned the form of hidden state representation: In order for a learner to adequately adapt one's beliefs in the face of changes to environmental statistics, more recent observations may be favored over past ones without modeling hidden state dynamics (Dirichlet-Categorical model; DC), or different sets of statistics may be estimated for a discretized latent state (Hidden Markov Model; HMM). Our comparison of these two learning approaches provides strong evidence for the DC model class over the HMM for the large majority of electrodes and post-stimulus time. The superiority of the DC model was found to be irrespective of the inclusion of leaky integration to the DC model, indicating the advantage of a non-hierarchical model in explaining the EEG data. Participants were neither aware of the existence of the hidden states in the data generation process, nor was their dissociation or any tracking of sequence statistics required to perform the behavioural task. As such, the early EEG signals studied here are likely to reflect a form of non-conscious, implicit learning of environmental statistics [84, 85, 86]. However, it is possible that the brain implements different learning algorithms in different environments, resorting to more complex ones only when the situation demands it. **As the discrete hidden states produced relatively similar observation sequences, more noticeable transitions between hidden states may provide an environment with greater incentive to implement a more complex model to track these states, which might have yielded different results.** Indeed, humans seem to assume different generative models in different contexts, possibly depending on task instructions [87]. This may in part explain why evidence has been provided for the use of both hierarchical [88, 89] and non-hierarchical models [90, 91]. **Nevertheless, it has been suggested that the brain displays a sensitivity to latent causes in low-level learning contexts (Gershman, Norman and Niv, 2015), which might indicate the relevance of other factors. For example, it is possible the currently tested HMM may be too constrained and a simpler, more general change-detection model [89] may have performed better.** By omitting instructions to learn the task-irrelevant statistics, our study potentially avoids the issue of invoking a certain generative model. We might therefore report on a 'default' model of the brain used to non-consciously infer environmental statistics.

Supplementary figure S7 subplot A:

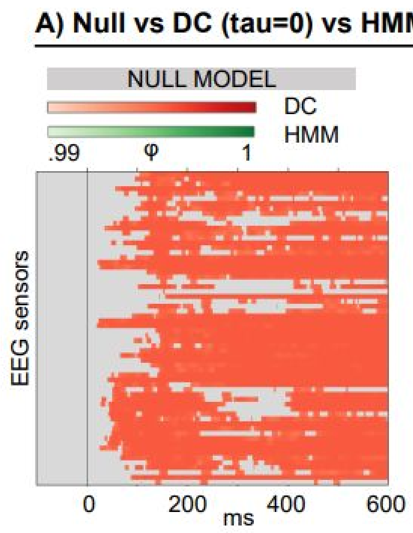

Additional random effects family-wise comparisons. A) Comparison of the model families: Null model, Dirichlet-Categorical model (DC) with tau = 0 (i.e. no forgetting and no penalization) and Hidden Markov Model (HMM). Exceedance probabilities (φ) are plotted for all comparisons.

- The authors fit the DC model leak parameter separately for each time bin of the evoked response, and found that the optimal parameter corresponding to early periods (where confidence-corrected surprise is encoded) differed from later periods (encoding Bayesian surprise). The authors also suggest that the former signal (CS) may control the latter (BS). Could the authors comment on how the difference in the time-scale of integration between the two signals is likely to affect this interaction.

We thank the reviewer for this comment. The hypothesis of early surprise signals controlling subsequent belief updating signals would indeed appear most straightforwardly compatible with the scenario in which both signals are computed using similar time-scales of integration. However, to the best of our knowledge, no mechanistic theory exists to date that specifies the relation of surprise signals and belief updating in the brain. The current investigation of the forgetting-parameter features some limitations, as touched on in the discussion. These include highly correlated regressors and considerable inter-individual variability leading to non-significance of a t-test between the optimized parameters for the early CS and subsequent BS signals. As such, the observed difference in best-fitting parameter values may result from the additional constraints of the surprise functions: Bayesian surprise converges to zero in the case of low forgetting and thus may be biased towards lower observation half-lives compared to predictive and confidence-corrected surprise. For these reasons we remain cautious in interpreting the difference in exact observation half-lives and instead consider the possibility that similar timescales of intermediary length underlie these signals. To clarify our interpretation, we have rephrased the relevant section in the discussion as follows:

Changes in the revised manuscript (l. 837):

Given a very large timescale, BS converges to zero as the divergence between prior and posterior distributions decreases over time, imposing an upper bound on the timescale. Meanwhile, for PS and CS it tends to lead to more accurate estimates of $p(y_t|s_t)$ as more observations are considered. However, given the regime switches in our data generation process, a trade-off exists where a timescale that is too large prevents flexible adaptation following such a switch. In the current context, the timescales are local enough where the estimated statistics are able to be adapted in response to regime switches (with a switch occurring every 100 stimuli on average). Especially CS shows a large range of τ-values producing similarly high model evidence due to the high correlation between regressors. **In sum, it is possible that the same timescale is used for the computation of both the CS and BS signals, as the differences in optimal τ-values between clusters were not found to be significant. This interpretation is most intuitively compatible with the hypothesis that the early surprise signals may control later belief updating signals.** Although the uncertainty regarding the exact half-lives is in line with the large variability found in the literature, local over global integration is consistently reported [13, 39, 90, 9, 48, 91]. Given a fixed inter-stimulus interval of 750ms, a horizon of 85 and 25 observations may be equated to a half-life timescale of approximately 63 to 17 seconds, with regime switches expected to occur every 75 seconds.

- The mathematical rigour with which the authors spell out their methods is commendable. However, for very simple points perhaps equations could be omitted to ease readability (e.g. equation 1 which simply reiterates that s(t) is 'static').

We thank the reviewer for their input. We agree the omission of the equation at line 246 is favourable for readability and removed it together with the accompanying sentence which spanned lines 247-248.

- Typo Figure 3 legend. Middle is the 'alternation probability' mode, not 'transition probability' model

We thank the reviewer for pointing out this unfortunate typo and have corrected it.

The figure caption (now Fig 2.) reads as follows:

Fig **2**. Dirichlet-Categorical model as a graphical model. Left: The stimulus probability model which tracks the hidden state vector determining the sampling process of the raw observations. Middle: The **alternation probability** model which infers the hidden state distribution based on alternations of the observations. Right: The transition probability model which assumes a different data-generating process based on the previous observations. Hence, it infers M sets of probability vectors α^i.

REVIEWER #2

I would happily support publication of this report in PLOS CB, as it offers both a new analysis framework to compare different models based on observed EEG responses, and has the potential to significantly advance our understanding of the mechanisms underlying somatosensory learning. However, I would challenge the authors to tap this potential further by being more explicit about (1) which learning mechanisms are supported/ruled out by their data, (2) what the different surprise signatures (PS, CS, BS) mean for an implementation of Bayesian learning, and (3) how the model-based results fit together with the MMRs identified in the conventional ERP analysis.

In particular, I would like to see the authors' response to the 4 main points listed below.

Sincerely,

Lilian Weber

Major:

1. First of all, I would challenge the authors with the following claim:

Showing that electrophysiological responses co-vary with specific computational quantities only contributes to a mechanistic understanding of the neuronal computations underlying the learning process, if

a) a concrete implementation of the computations that the quantity is involved in is conceivable (because the results can then be seen as prelim. evidence for such an implementation/neural process), or,

b) the specific quantities or the order of their representation rules out otherwise plausible proposals of the underlying mechanisms (i.e., not all variants of Bayesian inference in the somatosensory system are compatible with the observed pattern of results)

My feeling is that at least one of these is given in the current study, but would love to hear the authors' thoughts on this. I think this would greatly clarify the contribution that the current results make towards a mechanistic understanding of somatosensory learning.

We thank the reviewer for the insightful claim, which we would tend to agree with. Concerning a), although our study does not directly deal with the implementational level per se, the sort of computations (relating to surprise and belief updating in probabilistic inference) and learning models we consider are presumed to be compatible with popular theories of Bayesian brain function such as predictive coding and the free energy principle. For these, preliminary work suggests an implementational plausibility, e.g. on the level of cortical microcircuits (Bastos et al., 2012, Neuron, doi: 10.1016/j.neuron.2012.10.038). We propose our investigated models to share similarity with the concepts governing these theories and by extension may be subject to their claims of neural plausibility. Furthering the understanding of the concrete implementation of these learning models we consider of great importance for future research. Regarding b), we submit that a reverse order of the one found in the current study seems implausible (i.e. belief updating prior to surprise). However, despite early surprise signals having been reported, much of the literature focused on the P300 and it thus remained unclear whether early surprise signals only reflect puzzlement surprise or also encode belief updating. By considering two additional levels of model

comparison (concerning model class and sequence statistics), we not only aimed to contribute to the understanding of early somatosensory learning, but also to identify a more suitable generative model to further improve the ability to dissociate these quantities.

As in particular the topic of neurobiological plausibility goes unmentioned in the manuscript, we have added the followed text to the discussion section (l. 717):

By omitting instructions to learn the task-irrelevant statistics, our study potentially avoids the issue of invoking a certain generative model. We might therefore report on a 'default' model of the brain used to non-consciously infer environmental statistics. **The sort of computations (relating to surprise and belief updating) and learning models we consider might be viewed in light of theories such as predictive coding and the free energy principle for which preliminary work suggests implementational plausibility (e.g. Bastos et al., 2012). By extension, neural plausibility of the currently investigated models can be considered subject to their relation to such theoretical frameworks.**

In this context, I would also encourage the authors to carve out the critical difference between the two models they are comparing, to understand why the simpler model fits the data better. In their analysis approach the more complex model, which mimics the data generating process much better than the simpler DC model, is not penalized for complexity (because no subject-specific parameters are fitted). So what is the data feature that the DC-TP1 model captures, but the HMM-TP1 doesn't? E.g., does the HMM-TP1 predict different learning rates (and thus different surprise values) for the two different regimes (the volatile and the stable blocks), which are not supported by the data? Such insight would help to clarify the conclusions we can draw from the data about the learning mechanisms, and relate the results to the literature on whether or not participants adapt their learning rates to the volatility of the environment (e.g., refs 63, 86, 87, Behrens et al. 2007 Nat Neurosci).

The authors thank the reviewer for the interesting questions. The fast and slow switching regimes only refer to the transition probabilities between observations, while the probability for state transitions was fixed to p=0.01 across all blocks. As such, the current study is unfortunately not well suited to address the effects of the volatility of the environment on learning rates. We now comment on this interesting possible extension in our manuscript (please see below). In our interpretation, the main difference between the models is with respect to the HMM's attempt to dissociate the two latent states of the generative model, whereas they are combined/averaged for the DC.

We have chosen the following approach to improve model interpretation. First, we establish that the models provide distinct enough predictions so that they are recoverable under noisy conditions using our methodology in a simulation model recovery study. Subsequently, we have edited the regressor plots to better highlight the differences between models, and have added a description of these in the Methods section. Conducting further extended model interpretation analyses we consider out of the scope of the currently presented experimental work, but agree that future theoretical efforts in regards to this will be highly valuable.

Changes in the revised manuscript:

Discussion l. 769, regarding the volatility of the environment:

While PS is also a fast-computable puzzlement surprise measure and (similarly to CS) is scaled by the subjective probability of an observation, CS additionally depends on the confidence of the learner, read out as the (negative) entropy of the model. Evidence for a sensitivity to confidence of prior knowledge in humans has been reported in a variety of tasks and modalities (Boldt, Blundell, & De Martino, 2019; Meyniel & Dehaene, 2017; Payzan-LeNestour & Bossaerts, 2011). This further speaks to the possibility that CS informs belief updating, as confidence has been suggested to modulate belief updating for other modalities in the literature (Meyniel 2015; Meyniel 2020) and is explicitly captured in terms of belief precision by other promising Bayesian models (Mathys 2011; Mathys 2014). We suspect that, similarly, confidence concerns the influence of new observations on current beliefs in somatosensation. However, as this was not explicitly modelled and investigated in the current work we were not able to test it directly. **Furthermore, as the state transition probability between regimes was fixed in the current study, it is not well suited to address the effects of the volatility of the environment on belief updating. Future work might focus on the interplay of environmental volatility and confidence in their effects on the integration of novel observations.** It is important to note that one may also be confident about novel sensory evidence (e.g. due to low noise) which may result in larger model updates (Meyniel, Sigman, & Mainen, 2015). This aspect of confidence, however, lies outside the scope of the current work.

Model recovery methods addition (at the end of the methods section from l. 448):

A simulation model recovery study was performed to investigate the ability to recover the models given the sequence data, model fitting procedure, and model comparison scheme. To this end, data was generated for *n* = 4000 (corresponding to the five concatenated experimental runs) by sampling from a GLM $y \sim N(X\beta, \sigma^2 I_n)$, after which model selection was performed. For the null-model, the design-matrix only comprised a column of ones. For all non-null models, an additional column of the z-normalized regressor was added. We set the true, but unknown β_2 parameter to 1, while varying σ^2, which function as the signal and noise of the data respectively. Given the z-scoring of the data, the β_1 parameter responsible for the offset is largely inconsequential and thus not further discussed. The model fitting procedure was identical to the procedure described in the supplementary material used for the EEG analyses.

For each noise level, we generated 40 data sets (equaling the number of subjects) allowing for random-effects analyses. This process was repeated 100 times for each of the different comparisons: Null Model vs DC Model vs HMM (C1), DC TP1 vs TP2 (C2), DC SP vs AP vs TP1 (C3), and DC TP1 PS vs BS vs CS (C4). Family and model retrieval using exceedance probabilities worked well across all levels (S6 Fig 1A-D), with a bias to the Null Model as signal-to-noise decreases. By inspecting the posterior expected values of β_2 and λ^-1 which resulted from fitting the model regressors to the EEG data, an estimate of the signal-to-noise ratio that is representative of the experimental work can be obtained. By applying the thresholds of φ > 0.99, φ > 0.95, φ > 0.95, and φ > 0.9 across the four comparisons respectively and subsequently inspecting the winning families and models at σ^2=750 (i.e., an SNR of 1/750), no false positives were observed. For C1 and C4, recovery was successful for all true, but unknown models in all of the 100 instances. While for C2 and to a lesser extent C3, concerning the families of estimated sequence statistics, false negatives were

observed only when confidence-corrected surprise was used to generate data. For C2, this led to false negatives in 67 (TP1 CS) and 55 (TP2 CS) percent of cases, while for C3 28 (SP CS), 0 (AP CS), and 33 (TP1 CS) percent false negatives were observed.

Model recovery results supplementary figure:

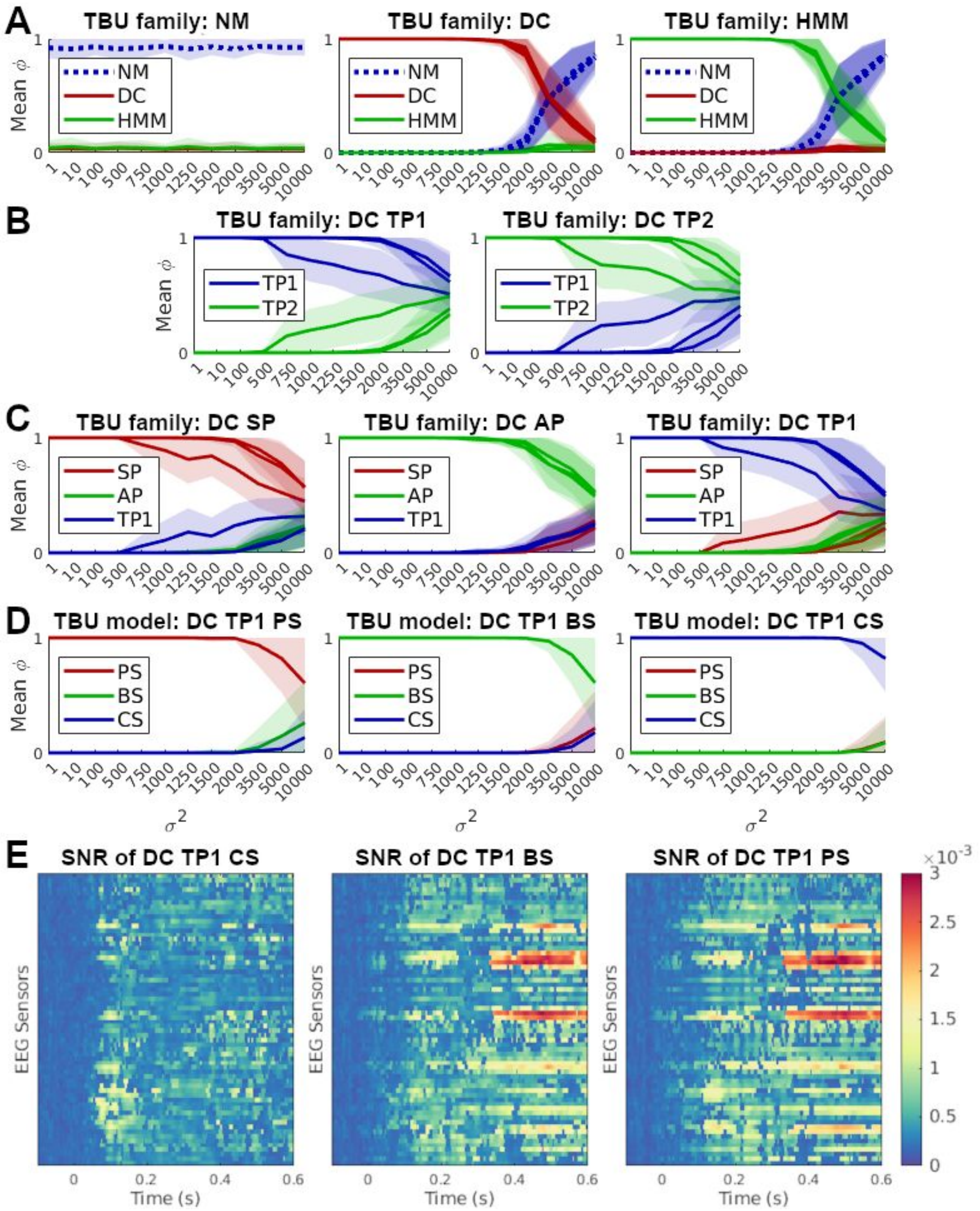

S6 Fig. Model recovery study. A model recovery study was performed using simulated data. Subplots A-D show the average exceedance probabilities (shading represents standard deviations) of 100 random-effects Bayesian model selection analyses under different signal-to-noise ratios. This was performed for (A) Null Model vs DC Model vs HMM families, (B) DC TP1 vs TP2 families, (C) DC SP vs AP vs TP1 families, and (D) DC TP1 PS, BS, and CS models. Noteworthy is that the instances of reduced differentiability for (B) and (C)

occurred only when the true, but unknown model was confidence-corrected surprise. (E) An estimate of the signal-to-noise of the experimental single-trial EEG analyses by inspecting the ratio of the expected posterior estimates of the model fitting procedure for β^2 and λ^-1.

Surprise regressor plot addition (replacing current figures 6 and 7)

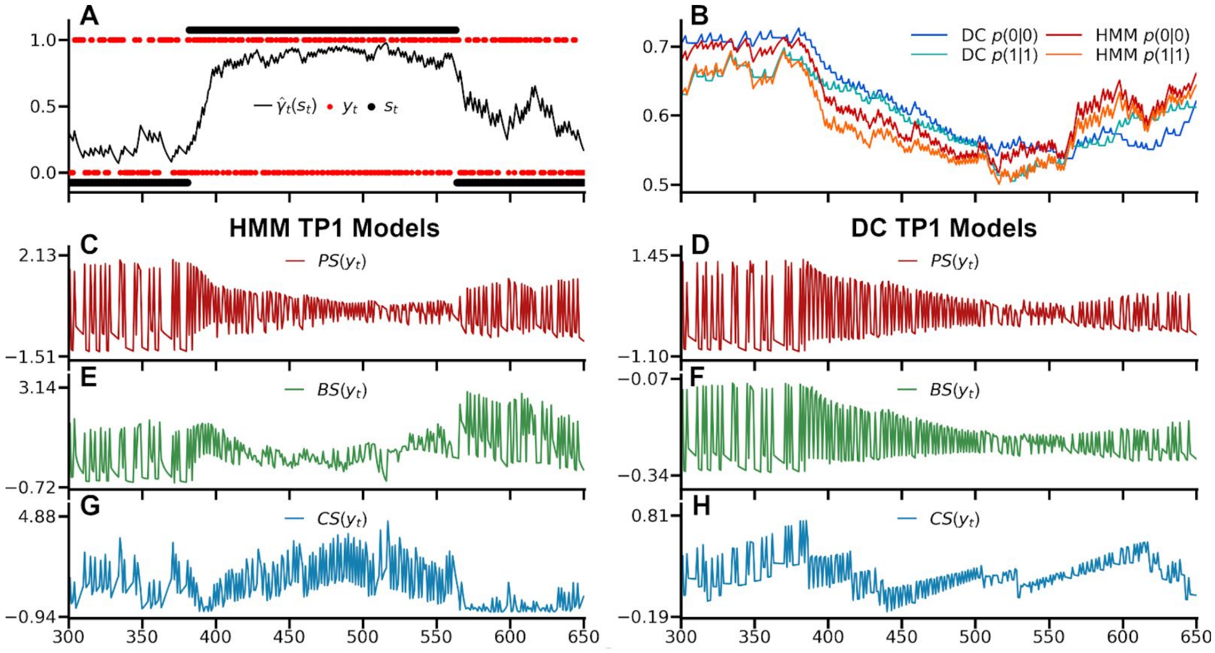

Fig 5. Surprise readouts. (A) Example sequence with o_t in red, s_t in black with s_t=0 for the slow-switching regime and s_t=1 for the fast switching regime, and the HMM filtering posterior $\hat{\gamma}(s_t)$ in between. The rare catch-trials are not plotted to facilitate a direct comparison between the HMM and DC models. (B) The normalized probability estimates of the HMM TP1 and DC TP1 model with an observation half-life of 95, displaying differences in estimates arising from different adaptations to regime switches. (C,E,G) The z-scored surprise readouts of the HMM TP1 models: predictive surprise (PS), Bayesian surprise (BS), and confidence-corrected surprise (CS). (D,F,H) The z-scored surprise readouts of the DC TP1 models.

Methods section addition describing the new plot to conclude the "Surprise readouts" section (l.354):

Fig 5 shows the regressors for an example sequence of the HMM TP1 and DC TP1 models with an observation half-life of 95. The PS regressors of both models show greater variability in the slow switching regime as compared to the fast-switching regime, where repetitions are more common (and consequently elicit less predictive surprise) while alterations are less common (and thus elicit greater surprise). As such, the PS regressors differ between regimes as a function of the estimated transition probabilities. The speed at which models adapt to the changed statistics depends on the forgetting parameter for the DC model while for the HMM it is dependent on the degree to which the regimes have been learned. BS is markedly distinct for the two models due to the differently modeled hidden state. DC BS features many small updates during the fast-switching regime, with more irregular, larger updates during the slow-switching regime, while HMM BS expresses the degree to which an observation produces changes in the latent state posterior. Finally, HMM CS is scaled by the confidence in the latent state posterior, tending to greater surprise the more committed the model is to one particular latent state, and lower surprise otherwise, such as at the end of

the example sequence. Meanwhile, due to its static latent state, confidence for DC CS results only from commitment to beliefs about the estimated transition probabilities between observations themselves, with rare events causing drops in confidence. Taken together, the HMM regressors ultimately depend on its posterior over latent states, and while this is absent for the DC, its regressors display differences between the two regimes as a function of its integration timescale which in turn allows it to accommodate its probability estimates to the currently active regime.

2. Secondly, one major claim of the study is that different measures of surprise are represented by EEG signals at different time points and sensors.

I would love to know what the authors think is the functional significance of PS/CS? In particular, in the update equations for the winning (DC) model, PS/CS is never used/computed explicitly. Why would the organism invest the additional energy to compute this (eqs.7,10,12), if it does not have any functional significance in updating beliefs? The authors, in the discussion, hint at a potential role of CS serving to control update rates (p.30, l.666), and interpret their findings as evidence that a higher-level region (S2) represents aspects of confidence, which is used to modulate belief updating on lower levels (S1). In other Bayesian models of inference learning like the HGF, update equations explicitly consider confidence (belief precision) as a driver of learning (update) rates. Such models have been used by our group to understand learning in auditory mismatch paradigms (Stefanics et al. 2018 J Neurosci; Weber, Diaconescu et al. 2020 J Neurosci). Do the authors see their data as compatible with such an account?

We indeed consider a role for puzzlement surprise in controlling rates of subsequent belief updating which may be regulated by belief confidence. However, we discuss this idea in relation to our data cautiously as our models do not prescribe a specific set of temporally ordered computations. That is to say, the currently tested models do not provide a plausible manner by which the brain acquires the estimated transition probabilities and subsequent surprise quantities. Rather, we view our model comparison as a methodology to infer on qualities that a future successful neural algorithm is likely to exhibit (e.g. using estimated transition probabilities to compute an early puzzlement surprise signal scaled by confidence). Insofar as confidence formulated as precision under the HGF and confidence as captured by confidence-corrected surprise both concern the balance between current beliefs and observations to inform belief updating, we suspect that these accounts are compatible. As we did not explicitly model and investigate the influence of confidence on updating of beliefs, as well as other differences between the models, it however remains an open empirical question.

Changes in the revised manuscript (Discussion: l.763):

While PS is also a fast-computable puzzlement surprise measure and (similarly to CS) is scaled by the subjective probability of an observation, CS additionally depends on the confidence of the learner, read out as the (negative) entropy of the model. Evidence for a sensitivity to confidence of prior knowledge in humans has been reported in a variety of tasks and modalities (Boldt, Blundell, & De Martino, 2019; Meyniel & Dehaene, 2017; Payzan-LeNestour & Bossaerts, 2011). **This further speaks to the possibility that CS informs belief updating, as confidence has been suggested to modulate belief updating for other modalities in the literature (Meyniel 2015, Meyniel 2020) and is explicitly captured in terms of belief precision by other promising Bayesian models**

(Mathys et al., 2011; Mathys et al., 2014). We suspect that, similarly, confidence concerns the influence of new observations on current beliefs in somatosensation. However, as this was not explicitly modelled and investigated in the current work we were not able to test it directly. Furthermore, as the state transition probability between regimes was fixed in the current study, it is not well suited to address the effects of the volatility of the environment on belief updating. Future work might focus on the interplay of environmental volatility and confidence in their effects on the integration of novel observations. It is important to note that one may also be confident about novel sensory evidence (e.g. due to low noise) which may result in larger model updates (Meyniel, Sigman, & Mainen, 2015). This aspect of confidence, however, lies outside the scope of the current work.

3. The authors present two complementary analysis approaches - a conventional average-based ERP analysis, and a single-trial model-based analysis. Both of these drive seemingly independent conclusions about the temporal dynamics of perceptual inference in peristimulus time: the results from the conventional analysis hint at early change detection in S1, then perceptual learning in S1/S2, and later attention-related effects. The results from the single-trial analysis suggest an early representation of CS in S2, and later representation of BS in S1. How do these relate to each other?

I would encourage the authors to address this question, for example by deriving predictions from the different models for MMR effects: (how) does the MMR arise from differences in surprise between trials labeled as standards and those labeled as deviants in the conventional analysis? What predictions do the models make about the effects of train length on surprise? Is the winning model compatible with the experimental observations for the different MMRs?

We thank the reviewer for the suggestion to attempt to more explicitly connect the two analysis approaches. We appreciate the input and agree that tying the different results together improves the manuscript. As such, we now provide a paragraph in the discussion where we relate them, starting at l. 797. (In order to avoid repetition we also removed a sentence at l.660)

Additional paragraph in the revised manuscript (l. 797):

Conjointly, the average-based ERP analysis shows an early MMR indicative of change-detection around 57ms in S1, while the single-trial analysis indicates CS encoding from around 70ms in S2. Given the temporal and spatial difference, these may correspond to different responses. On the other hand, both the early 57ms MMR as well as CS as a surprise quantification share an apparent independence of their response to train lengths. Namely, PS and BS decrease as a stimulus is repeated and are proportionally greater in response to a deviant. CS is scaled by PS and BS, as well as by belief commitment, which increases for standards and decreases for deviants. This counteracting effect of belief commitment and the surprise terms can lead to independence of CS and train length when responses are averaged, as appears to be the case for the early MMR, indicating the possibility for a potential relation between these results. The intermediate MMR roughly temporally co-occurs with a simultaneous representation of BS and CS in S1 and S2. The dependence of the mid-latency MMR on train-length for both standards and deviants and the encoding of belief inadequacy and updating quantities is suggestive of convergent support in favor of a perceptual learning response which involves both somatosensory cortices.

Finally, the P300 MMR spans both late BS clusters, indicating a role of the P300 response in Bayesian updating, which has been previously reported (Ostwald et al., 2012, Kolossa et al., 2015), and might specifically reflect an updating process of the attention-allocating mechanism as suggested by Kopp and Lange (2013).

4. Separate, independent model comparisons are performed for each sensor and peristimulus time bin. (As far as I can tell, the variational inference procedure described in the supplementary section S2 was applied on all of these data points separately.) Can the authors comment on whether this creates a multiple comparison problem and if yes, in how far their analysis deals with this? Does their choice of exceedance probabilities at each step of the hierarchical model comparison, and/or their choice of cluster size thresholds (in time and sensor space) used for detection of significant clusters account for this?

In Bayesian model comparison there is no conventional way to correct for multiple comparisons and it has been established that Bayesian methods provide inherent adjustments of sensitivity and specificity to deal with false positive rates (Friston 2002, Neuroimage, doi:10.1006/nimg.2002.109 and Friston 2002, Neuroimage, doi:10.1006/nimg.2002.109). However, in line with a comment of reviewer #3 (below) we added information on the number of comparisons in the text.

Also, to get an impression of the model fit beyond the relative comparison to other models, can the authors report the % variance explained in the trial-by-trial EEG amplitudes by the winning model?

The authors thank the reviewer for their suggestion. The percent variance explained (PVE) in the data by the winning models is between 0.1 and 0.4 % for both the EEG as well as the dipole amplitudes. While the explained variance is quite small, comparable reports of kindred studies are largely lacking. However, the reported range of PVE is in agreement with a recent MEG study using surprise regressors of comparable Bayesian models (Maheu et al., 2019, eLife, doi: 10.7554/eLife.41541). In order to provide this information in the manuscript we edited the presentation of the dipole model comparison results (Fig. 13) to include the PVE by the respective regressors.

Changes in the revised manuscript:

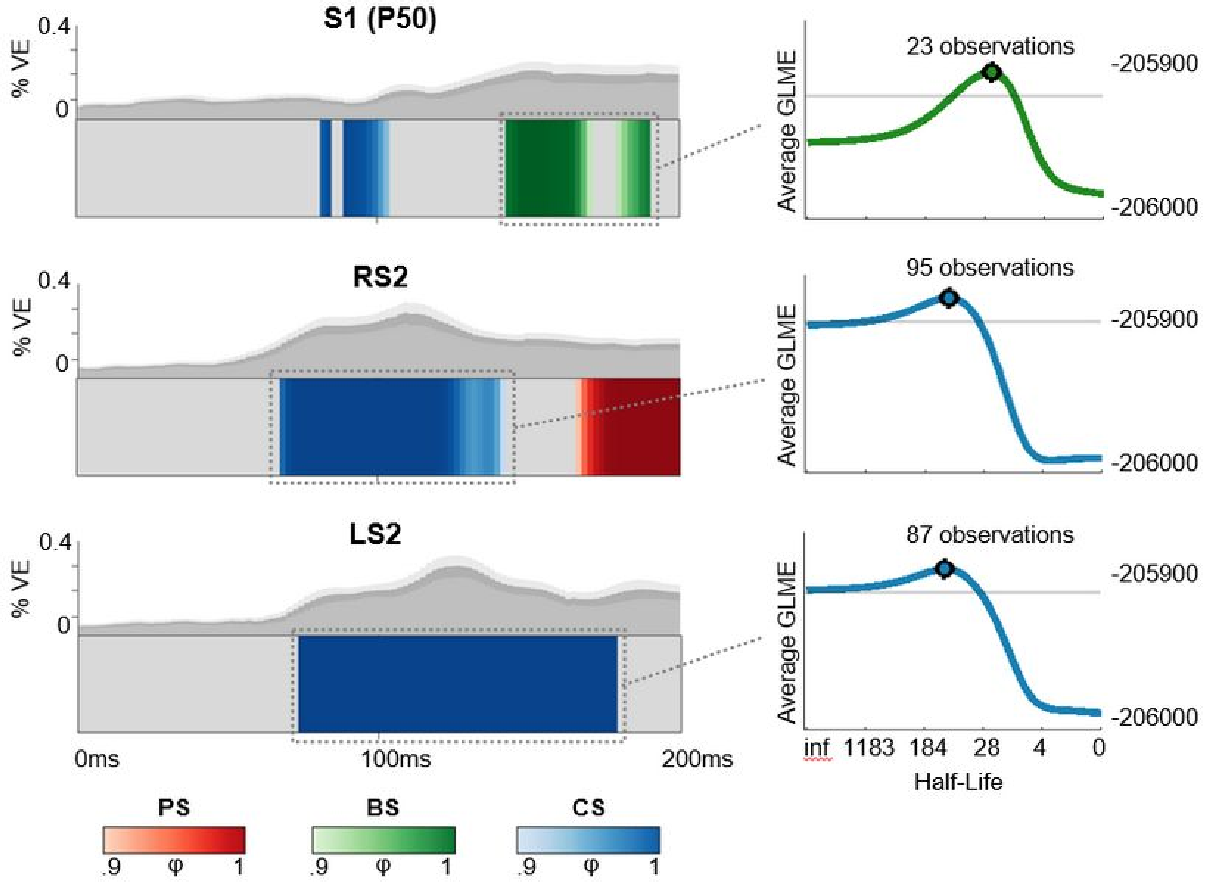

Fig. 11. Modeling results in source space with best fitting forgetting-parameter values. Red: Predictive surprise (PS), Green: Bayesian surprise (BS), Blue: Confidence-corrected surprise (CS) A) Colors depict significant time points for the surprise readout functions of the Dirichlet-Categorical TP1 model within the dipoles S1P50, right S2 (RS2) and left S2 (LS2). The S1N20 dipole was omitted in the visualization as no significant effects were observed. Grey area plots above each dipole plot show the respective mean percent variance explained of the winning models +- standard error. Thus, the variance explained of BS (S1P50 dipole) and CS (RS2 and LS2 dipoles) is plotted. B) The group log model evidence (GLME) values corresponding to the stimulus half-lives for forgetting-parameter τ, after averaging the significant timebins of the dipoles (S1P50: 145-191ms; RS2: 68-143ms; LS2: 76-168ms). The grey lines indicate a difference of 20 GLME from the peak, indicating very strong evidence in favour of the peak half-life value compared to values below this threshold.

Minor comments/questions:

Intro:

- p.3,l.66: I find the reference to prediction error confusing here, as (precision-weighted) PE in Bayesian models is often equivalent to model adjustment (Bayesian surprise)

To avoid any potential confusion we have removed this reference to prediction error.

- p.3,l.72 etc.: the introduction of the different surprise measures could be improved. First if all, predictive (Shannon) surprise in practical applications (including here) is computed with reference to subjective beliefs about the probability of events, not the objective frequency. Second, the difference to CS then remains vague, and the mathematical description for CS which is given on p.15 comes very unexpected. Can the authors more clearly state in the introduction what is different in CS from PS (e.g., even if an event is subjectively unlikely (PS), it is not necessarily surprising)?

The authors thank the reviewer for pointing out helpful clarifications of the definitions of surprise to highlight their differences in the introduction and prepare the reader for their mathematical definitions. The respective paragraph starting at l.44 has been edited with this in mind:

In the context of probabilistic inference, the signalling of a mismatch between predicted and observed sensory input may be formally described using computational quantities of surprise [6, 34]. By adopting the vocabulary introduced by Faraji et al. [35] surprise can be grouped into two classes: puzzlement and enlightenment surprise. Puzzlement surprise refers to the initial realization of a mismatch between the world and an internal model. **Predictive surprise (PS) captures this concept based on the measure of information as introduced by Shannon [36]. Specifically, PS considers the belief about the probability of an event such that the occurrence of a rare event (i.e. an event estimated to have low probability of occurrence) is more informative and results in greater surprise. Confidence-corrected surprise (CS), as introduced by Faraji et al. [35] extends the concept of puzzlement surprise by additionally considering belief commitment. It quantifies the idea that surprise elicited by events depends on both the estimated probability of occurrence as well as the confidence in this estimate, with greater confidence leading to higher surprise. For example, in order for the percept of a drop of rain on the skin to be surprising, commitment to a belief about a clear sky may be necessary.**

Methods:

- p.5, l.118: 'oddball-like'?

The formulation of an oddball-like roving-stimulus paradigm was referring to the similarity of our (roving-stimulus) paradigm with the classic oddball paradigm (which most readers will be familiar with) while highlighting that the roving-stimulus paradigm differs in important ways. In

an attempt to avoid any confusion we are now simply stating to use a “roving-stimulus paradigm”.

- p.7: It would be much easier for the reader to first briefly describe the resulting tone sequence and then go into the generative model for it.

The generation of stimuli sequences (p.7) follows the description of the sequence presentation (p.5) where an exemplary part of a sequence is plotted and described. To further combine the generative model and resulting sequence properties we now present one combined figure (now Fig. 1) including previous Fig. 1 and Fig. 2 as well as additional information on the average train lengths per regime as requested in the comment below.

Changes in the revised manuscript (Fig. 1):

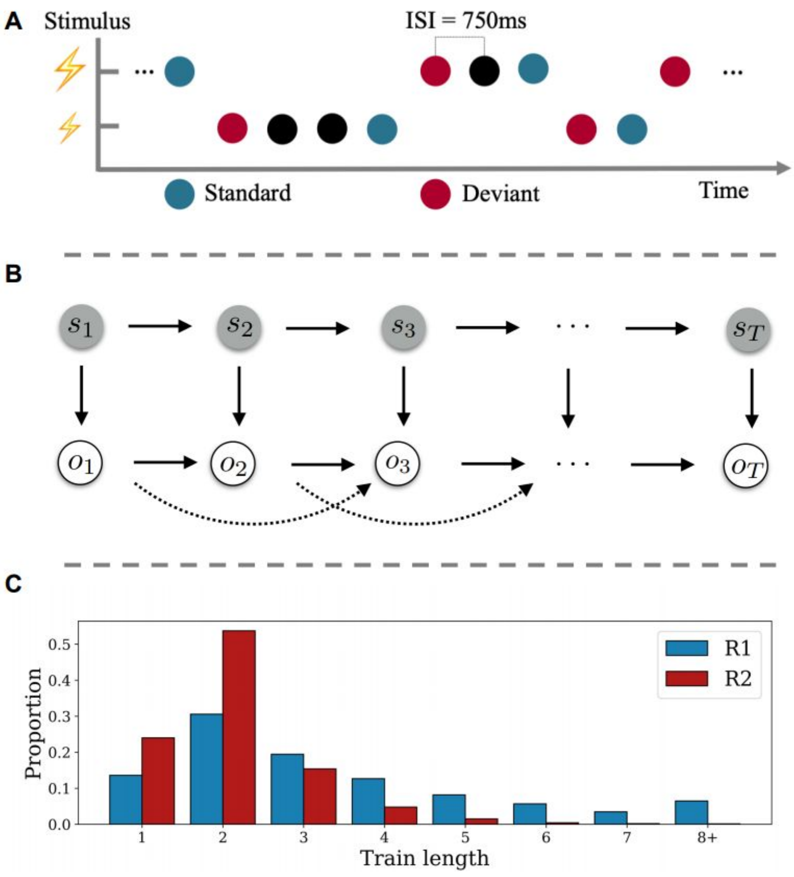

Fig. 1. Experimental design and stimulus generation. A) Presentation of experimental stimuli using a roving-stimulus paradigm. Stimuli with two different intensities are presented. Their role as standard or deviant depends on their respective position within the presentation sequence. B) Graphical model of data-generating process. Upper row depicts the evolution of states s_t over time according to a Markov chain. The states emit observations o_t (lower row), which themselves feature second order dependencies on the observation level. C) Average proportion of resulting stimuli train lengths. Higher proportion of shorter trains for the fast switching regime (R2;

red) and more distributed proportion across higher train lengths for the slow switching regime (R1; blue).

- p.8, table1: please provide stimulus stats, e.g. average train length in the two regimes

We thank the reviewer for the suggestion. We have now added a plot (Fig. 1C - please see the previous comment) which details the average proportion of train lengths in each regime.

- p.8, 'Event-related potentials' - given that the GLM already included the parametric regressors for train length, why was this effect further investigated in the significant beta estimates by testing for a linear relationship with train lengths?

The reported linear fit of train lengths for standards and deviants is equivalent to the results of the GLM parametric contrast and shows the same p-values at the peak voxel (of the main effect, standards vs. deviants). Since we are interested in the details of these main effects we reported only these corresponding betas and used linear fits for the convenience of plotting the regression lines for visualization purposes. To be more explicit the revised manuscript l.182-185 now reads:

The significant peaks of the GLM were further inspected by looking at their effect of train length and the corresponding β-parameter estimates of each train length were subjected to a linear fit for visualization purposes.

- p.11-13: a simple and intuitive description of the DC model learning process might be given, e.g. 'the observer simply counts the observations of each type to determine her best guess of their probability (eq.6), with an exponential forgetting, i.e. discounting observations the further in the past they occurred (eq.9).'

We thank the reviewer for this helpful suggestion to provide a more intuitive description. We appreciate the suggestion and added the following text to the DC model section (l.253):

The Dirichlet-Categorical model is a simple Bayesian observer that counts the observations of each unique type to determine its best guess of their probability (eq. 5). Its exponential forgetting parameter implements a gradual discounting of observations the further in the past they occurred (eq. 8).

- it seems from figure 6 that catch trials were included for the DC model? If so, why were they modeled for one model, but not the other (HMM)?

With the intent to model the sequence data as complete as possible, catch trials were indeed included for the DC model (though as described, the trials were deleted prior to model fitting). To facilitate the HMM implementation, the catch trial regime was omitted as it was not a hidden state (catch trials are emitted with a probability of 1). To make sure that the superiority of the DC model is not due to the modelling of catch trials, we re-analyzed the

Null Model vs DC vs HMM family comparison without modeling the catch trials for the DC model, which still showed a clear superiority of the DC model without any significant results for the HMM (see S7 Fig. B). Additionally, we inspected the effect of modelling the catch trial with the DC model in a family comparison of Null vs DC-catch vs DC-no-catch (see S7 Fig. C) which shows that a complete DC model that includes all observations of the participants is a better fit than a less complete DC model.

Supplementary figure addition in revised manuscript:

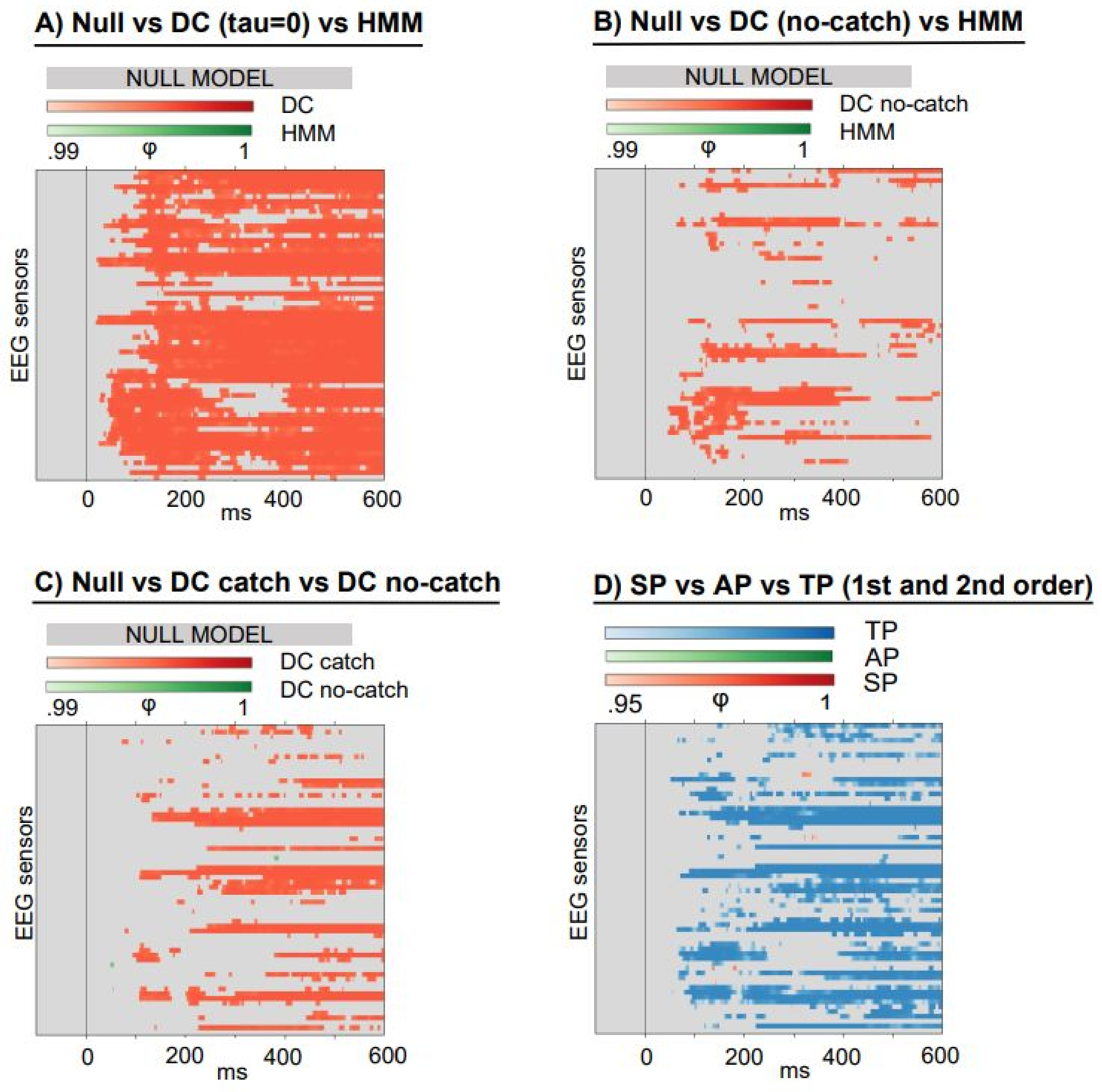

S7 Fig. Additional random effects family-wise comparisons. A) Comparison of the model families: Null model, Dirichlet-Categorical model (DC) with tau = 0 (i.e. no forgetting and no penalization) and Hidden Markov Model (HMM). B) Comparison of the model families: Null, DC without modelling the catch trial and HMM. C) Comparison of the model families: Null, DC with and DC without modelling the catch trial. D) Comparison of the model families within the DC model: Stimulus probability model (SP), alternation probability model (AP) and transition probability model family (TP) subsuming first and second order TP models in one family. Exceedance probabilities (φ) are plotted for all comparisons.

- in addition to visualizing the surprise readouts, it would be nice to also visualize the learning process itself, in particular in the DC model (e.g. the evolution of the estimated probability vector alpha over the tone sequence) - a figure for the DC model similar to fig.5 for the HMM.

As described in response to a comment above, in order to include a better representation of the behaviour of the models we present a new combined figure (Fig. 5) including exemplary trials of a sequence and the corresponding behaviour of all model regressors as well as the state estimation of the HMM. This figure now also includes the evolution of the estimated transition probabilities over a sequence for both the DC and HMM.

- p.14, l.327-334: This is not clear. In particular, l. 333 "Thus, the HMM estimates two vectors of emission probabilities corresponding to these events" - which two vectors and which events?

Given that the HMM estimates emission probabilities of the form $p(o_t|s_t)$, it was necessary to re-code all observations to reflect whether an alternation or repetition occurred (AP) or which transition occurred (TP), as the identity of these events depend on o_{t-1} as well as s_t. A two-state HMM estimates two sets (rather than our previous use of 'vectors') of emission probabilities of such events.

We have adjusted the text in order to improve clarity (l.314):

The aim of the HMM was to approximate the data generation process more closely by using a model capable of learning the regimes over time and performing latent state inference at each timestep. To this end, prior knowledge was used in its specification by fixing the state transition matrix close to its true values ($p(s_t = s_{t-1}) = 0.99$). The rare catch trials were removed from the data prior to fitting the HMM and thus their accompanying third regime was omitted, resulting in a two-state HMM. Given that an HMM estimates emission probabilities of the form $p(o_t|s_t)$ and thus does not capture any additional explicit dependency on previous observations, the input vector of observations was transformed prior to fitting the models. For AP and TP inference this equated to re-coding the observation o_t to reflect the specific event that occurred. Specifically, for the AP model the input sequence was $dt = 1_{o_t \neq o_{t-1}}$, while for TP1 and TP2 a vector of events was used corresponding to the four possible transitions from o_{t-1} or eight transitions from o_{t-2} respectively. **Thus, the HMM estimates two sets (reflecting the two latent states) of emission probabilities which correspond to the events (y_t).** Despite this deviation of the fitted models from the underlying data generation process, the AP and TP models reliably captured R1 and R2 to their capability, with TP2 retrieving the true, but unknown underlying emission probabilities (see **S3 Fig**). As expected, SP inference was agnostic to the regimes, while AP and TP inference allowed for the tracking of the latent state over time (**S3 Fig**). An example of the filtering posterior may be found in Fig **4**.

- p.15, figure 5: might be worth mentioning that the 2 states modelled by the SP model do not correspond to the two regimes - the figure might suggest that p(s_t) should track the underlying regimes, while s_t has a different meaning for the SP!

We thank you for your helpful suggestion. This is indeed correct. To prevent any such confusion, we have edited the caption of Fig 4 (was Fig 5):

Fig 4. Posterior probabilities of the HMM. Comparison of the posterior $p(s_t|o_1, ..., o_t)$ of the different HMM inference models for an example sequence. The true, but unknown regimes of the data generation process are plotted in red. **Note that, as the regimes were balanced in terms of stimulus probabilities, SP inference is not able to capture the underlying regimes and instead attempts to dissociate two states based on empirical differences in observed stimulus probabilities.**

- p.15, l.355: the prior used in CS is not the (flat) prior of the naive observer: CS = KL between the informed prior and the naive posterior.

Thank you for bringing this unclear phrasing to our attention. The text on l.337 now reads:

"It is defined as the KL divergence between the **informed** prior and the posterior distribution of a naive observer, where the naive posterior corresponds to a flat prior $\hat{p}(s_t)$ (i.e. all outcomes are equally likely) which observed y_t."

- p.17: might be worth mentioning that regressors were the same across participants (or if they differed, they only did so because the stimulus differed), and no participant-specific parameters were estimated (except for the optimization of tau)

Thank you for this suggestion. l.382 now reads:

"Each combination of model class (DC and HMM), inference type (SP, AP, TP1, TP2), and surprise readout function (PS, BS, CS) yields a stimulus sequence-specific regressor. **The same models were used across subjects and as such the regressors did not include any subject specific parameters.** These regressors, as well as those of a constant null-model, were fitted to the single-trial, event-related electrode and source activation data."

- p.17 please state the total number of linear regressions run (i.e., number of sensors x number of peristimulus time bins) (i.e., the total number of model comparisons run for 'independent' data points)

- p.18, l.415: and each sensor?

We have now clarified this in the text (l.422):

The furnished model evidences were subsequently used for a random-effects analysis as implemented in SPM12 [61] to determine the models' relative performance in explaining the EEG data. In order to combat the phenomenon of model-dilution [65], a hierarchical

approach to family model comparison was applied (for a graphical overview see S3 Fig). Note that this procedure is performed for each peri-stimulus time bin **and electrode** independently **(resulting in 22976 model comparisons per subject)**. In the first step, the two model classes DC and HMM were compared against each other and the null-model in a family-wise BMS. A threshold of exceedance probabilities φ>0.99 in favour of either the DC or HMM was applied, so that only whenever there was very strong evidence in favour of one of the model classes the following analyses were applied. For timepoints with exceedance probabilities above this threshold, a family-wise comparison of TP1 and TP2 was performed in order to determine which order of transition probabilities would be used for the second level. Subsequently, either the TP1 or TP2 models were compared to the SP and AP models. Wherever φ>0.95 for one of the inference type families, the third analysis level was called upon. On this final level, surprise read-out functions were compared for the winning model class and corresponding inference type with a threshold of φ>0.9. As such, this step-wise procedure allows spatio-temporal inference of the read-out functions for which there is strong evidence of the belonging model class and inference type. The same procedure was used for the EEG sensor and source data.

Results:

- p.23, l.490&491: exact p-values and t statistic?

We are happy to provide these statistics and have included them in the section, it now reads (l.541):

“The S1P50 dipole shows a significant difference at both time windows (at 57ms **p=0.006, t=2.94**; at 119ms **p=0.009, t=2.75**; bonferroni corrected) and can be suspected to be the origin of the effect at 57ms as well as contribute to the 119 MMR while the right S2 dipole is mainly driving the strong 119ms effect (at 119ms **p=0.001, t=3.44**; bonferroni corrected).”

- p.23, l.510-512: please explain this here, so that the reader does not have to refer to the supplementary to understand what is plotted in the scalp topographies. Please state explicitly in the text and the figures what the scalp topographies show, and what this parameter means.

To aid in interpreting the parameter and its topographies, we have added context to its meaning in the figure caption, while removing it’s mention in the text itself:

Change in the revised manuscript:

Fig 10D shows the result of the random-effects Bayesian model selection analysis. The scalp topographies depict the winning readout functions of the Dirichlet-Categorical TP model at different time windows. ~~**The converged variational expectation parameter m resulting from the model fitting procedure (see S2 Appendix) are displayed for the winning models to facilitate interpretation of the topography.**~~ Given the difference in temporal dynamics of faster, early (<200 ms) and slower, late (200-600ms) EEG components, different thresholds were applied. Early significant clusters were identified by averaging exceedance probabilities over 10ms windows and using a minimum cluster size of two electrodes. After 200ms, clusters were identified by averaging over 50ms time windows

with a minimum cluster size of four. From around 70ms on, early surprise computations can be observed with confidence corrected surprise (CS) best explaining the EEG data on contralateral and subsequently ipsilateral electrodes up to around 200ms. A significant cluster of Bayesian surprise (BS) is prominent at centro-posterior electrodes between 130-200ms, with similar electrodes later again representing Bayesian surprise around 300 and 375ms. These clusters are temporally in accordance with the N140 and P300 MMR effects. The latest cluster at around 500ms post-stimulus is entirely driven by predictive surprise.

Model caption change in the revised manuscript

Fig **10**. Modeling results. Exceedance probabilities (') resulting from the RFX family model comparison. A) Dirichlet-Categorical (DC) model, Hidden Markov Model (HMM) and Null model family comparison, thresholded at '>0.99:. B) Family comparison within the winning DC family, thresholded at φ>0.95: first and second order transition probability models (TP1, TP2). C) Family comparison within the winning DC family, thresholded at φ> 0:95: first order transition probability (TP1), alternation probability (AP) and stimulus probability (SP) models. D) Family comparison of surprise models within the winning DC TP1 family, thresholded at φ >0.9: Large discrete topographies show the significant electrode clusters of Predictive surprise (PS) in red, Bayesian surprise (BS) in green and confidence-corrected surprise (CS) in blue. Small continuous topographies display the converged variational expectation parameter m_β. **This parameter may be interpreted as a \beta weight in regression, indicating the strength and directionality of the weight on the model regressor that maximizes the regressor’s fit to the EEG data (see S2 Appendix).**

- figure 12 and figure S3: please state explicitly which steps resulted in data reduction (i.e., a selection of EEG sensors and time points for which a meaningful model comparison results could be retrieved, to be included in the comparison at the next step)

Currently, the steps for data reduction are explained in the methods section headed ‘Bayesian model selection’. We have now edited the figure descriptions to include this information.

Changes in the revised manuscript:

Fig 10. Modeling results. Exceedance probabilities (φ) resulting from the RFX family model comparison. A) Dirichlet-Categorical (DC) model, Hidden Markov Model (HMM) and Null model family comparison, thresholded at φ>0.99 **and applied for data reduction at all further levels.** B) Family comparison within the winning DC family,thresholded at φ>0.95: first and second order transition probability models (TP1, TP2) determining which order of TP is compared to stimulus probability (SP) and alternation probability (AP) models. C) Family comparison within the winning DC family, thresholded at φ > 0.95 **and applied at the final level.** D) Comparison of surprise models within the winning DC TP1 family, thresholded at φ > 0.9: Large Discrete topographies show the significant electrode clusters of Predictive surprise (PS) in red, Bayesian surprise (BS) in green and confidence-corrected surprise (CS) in blue. Small continuous topographies display the converged variational expectation parameter m_β. This parameter may be interpreted as a β weight in regression, indicating the

strength and directionality of the weight on the model regressor that maximizes the regressor's fit to the EEG data (see S2 Appendix).

S4 Fig. Hierarchical approach to family wise Bayesian model selection. First level (depicted in the top row): The 12 DC models and the 12 HMM models were grouped into their corresponding model class family and compared via BMS against each other and an offset Null-Model. Second level (lower row, left rectangle): Within the DC model class, the two transition probability models $TP_1$and TP_2 were grouped into families and the winner of the BMS was used for the comparison against the other two inference type models (Stimulus Probability (SP) and Alternation Probability (AP)). Third Level (lower row, middle rectangle): The surprise readouts of the DC TP_1 model were subjected to BMS and the resulting exceedance probabilities are reported in the main results. **Thresholding of the model class families and inference types was again applied at successive levels leading to data reduction.**

- figure 13: please state the unit for the half-life (observations?)

The unit for half-life is indeed observations and is now stated as such in the figure.

Discussion:

- p.26, l.548-550: the interpretation, especially of the P300 effect as an attention-allocating process, comes somewhat ad-hoc, because it hasn't been motivated before. It is discussed later again (p.27, l.580-6), but only afterwards (p.28) are the aspects of the current results mentioned which support this interpretation (i.e., linear dependence of the P300 to deviants on train length). If this is the main finding that the authors base their attentional interpretation on, it should be mentioned earlier.

We appreciate the concern and present our interpretation now later in the discussion. l.623 now reads:

"**The early MMR effects were source localized to the somatosensory system and the N140 and P300 MMR's show differential linear dependence on stimulus train lengths for standard and deviant stimuli.** Using computational modelling, …"

- p.29, l.622-625: Not sure I understand this conclusion. The winning model did not learn about the different regimes in any way, so neither explicit nor implicit learning of the regimes are supported.

Here we intend to refer to the more general forming of probability estimates of observations, rather than discrete hidden state inference While the DC model is not able to explicitly capture any volatility in the environmental statistics (i.e. our regime change probability) it is however able to approximate the sequence statistics across the regimes by utilizing exponential forgetting to account for change-points as we discuss in the following section of

the discussion. We do appreciate that this is currently not obvious given the preceding sentence happens to only concern the hidden states.

We try to be more clear with the following changed text (l.705):

“Even though the data generation process included discrete hidden states in the form of fast and slow switching regimes, participants were neither aware of their existence nor was their dissociation **or any tracking of sequence statistics** required to perform the behavioural task. As such, the early EEG signals studied here are likely to reflect a form of non-conscious, implicit learning of environmental statistics.”

- p.29, l.630: one of the cited studies (ref. 86) employed a very different form of hierarchy without an explicit representation of change points. This model (the HGF) is actually more similar to the non-hierarchical DC model used here, except that the leakiness (learning rate) is a function of a subjective estimate of volatility (i.e., continuous rate of change).

Although it was the intention to distinguish between models that include or exclude some hierarchical representation of change-points and/or volatility in general, the reviewer’s point is well taken and in response we have exchanged referenced #86 with Behrens et al., 2007 (doi:10.1038/nn1954) as an alternative example of an hierarchical learning model.

REVIEWER #3

Major points:

Hierarchical model comparison approach: You have chosen to act against the "dilution of evidence" across many models by invoking a hierarchical model comparison scheme based on exceedance probabilities in a series of hierarchical comparisons. While there are other examples of such a hierarchical approach in the literature, this is, to my knowledge, not standard in family comparison of models, and I am not aware of any paper that suggests that this procedure is correct for selecting the best model. Every model or family comparison is conditional on the model space that you put in. In the extreme case, your final set of three models that you compare might not even include the best of all models. I would recommend running a model comparison over all models and running three family comparisons where you arrange your families to compare models along the three dimensions on your model space. Even if model comparison turns out to be inconclusive, this is an important information for the reader and the family comparisons should allow you to make some general statements about the different dimensions of your model space, which are of interest. In conclusion, I think that using the hierarchical scheme, you cannot safely conclude that "EEG signals were best described using a non-hierarchical Bayesian learner performing transition probability inference." But, maybe the search for a single best model is not even the most important goal here if you can make more robust and solid statements about other dimensions, e.g. whether an HMM or DC is better, or which kind of Surprise explains the data best at what time point, irrespective of the precise formulation of the other aspects of the model.

We thank the reviewer for the detailed consideration of our approach. We acknowledge that the description of our BMS procedure might have been confusing. First, we would like to clarify that the approach employed here is not a hierarchical Bayesian analysis per se but rather a step-wise procedure of dimensionality/data reduction, reminiscent of a strategy also applied in dynamic causal modeling. The order of our step-wise model comparison is motivated by the internal 'hierarchy' of our model space (further detailed below) and the concern for interpretability of the results. Specifically, in case of a non-hierarchical family comparison situations may arise where for specific EEG sensors and time points significant results are found for the surprise readout functions, but it is unclear what generative model underlied these computations. Vice versa, we considered it problematic to provide results of a certain generative model but not being able to comment on which computations this model appeared to perform. As such, we opted for a step-wise procedure that for a specific EEG sensor/timepoint allows for an interpretation of the model class (HMM or DC model), which sequence statistics this model tracked (stimulus, alternation, or transition probability), and finally which surprise computation is performed.

We decided to additionally include an evaluation of the random effects analysis in the manuscript by reporting a simulation study to validate model recovery across the different levels of the hierarchical model comparison approach. The details of the simulation are described in our response to reviewer #2 above and are an addition to the methods section. Together with the replies to specific concerns below we hope to show that our approach is suited for selecting the model that best explains the observed EEG data.

Nevertheless, we appreciate the reviewer's interest in the outcome of an alternative approach to the applied hierarchical BMS steps and thus included results of the suggested

non-hierarchical model comparison in the supplementary material (S5 Fig.). In order to get an idea of the best fitting model for the estimation of the sequence statistics, the non-hierarchical approach partitions the full Null, DC and HMM model-space into Null, SP, AP, TP1 and TP2 model families. The results indicate that the first order transition probability model is again the winning model across most electrodes and time points. However, one can observe that the effects are more pronounced for the later time points as a result of the model dilution of TP1 and TP2 (CS) prior to 200ms. The issue of comparing TP1 and TP2 (relating also to a different question of the reviewer) is addressed separately below. Furthermore, the non-hierarchical comparison of surprise (resulting from partitioning the full model space into PS, BS and CS families) results in a highly similar pattern of results to the reported hierarchical approach. The electrode topography shows the same clusters of CS and BS prior to 200ms as well as weaker later clusters of BS and PS (the latter observation is also found in the hierarchical comparison and is further addressed below).

Changes in the revised manuscript (l. 420 and from l.438 onwards):

The furnished model evidences were subsequently used for a random-effects analysis as implemented in SPM12 (Stephan et al., 2009) to determine the models' relative performance in explaining the EEG data. In order to combat the phenomenon of model-dilution (Penny et al., 2010), a hierarchical approach to family model comparison was applied (for a graphical overview see S4 Fig.). **This amounts to a step-wise procedure that leads to data-reduction at subsequent levels.** Note that this procedure is performed for each peri-stimulus time bin independently. In a first step, the two model classes DC and HMM were compared against each other and the null-model in a family-wise BMS. A threshold of exceedance probabilities $\varphi > 0.99$ in favour of either the DC or HMM was applied, so that only whenever there was very strong evidence in favour of one of the model classes the following analyses were applied. For timepoints with exceedance probabilities above this threshold, a family-wise comparison of TP1 and TP2 was performed in order to determine which order of transition probabilities would be used for the second level. Subsequently, either the TP1 or TP2 models were compared to the SP and AP models. Wherever $\varphi > 0.95$ for one of the inference type families, the third analysis level was called upon. On this final level, surprise read-out functions were compared for the winning model class and corresponding inference type with a threshold of $\varphi > 0.9$. As such, this step-wise procedure allows spatio-temporal inference of the read-out functions for which there is strong evidence of the belonging model class and inference type**, facilitating the interpretation of the results. The hierarchical ordering thus moves from general to specific principles: the model class and inference type determine the probability estimates of the model, which are finally read out through surprise computation. As a control analysis and inspection of the effect of our hierarchical scheme, we performed a non-hierarchical family comparison analysis (S5 Fig).** The same procedure was used for the EEG sensor and source data.

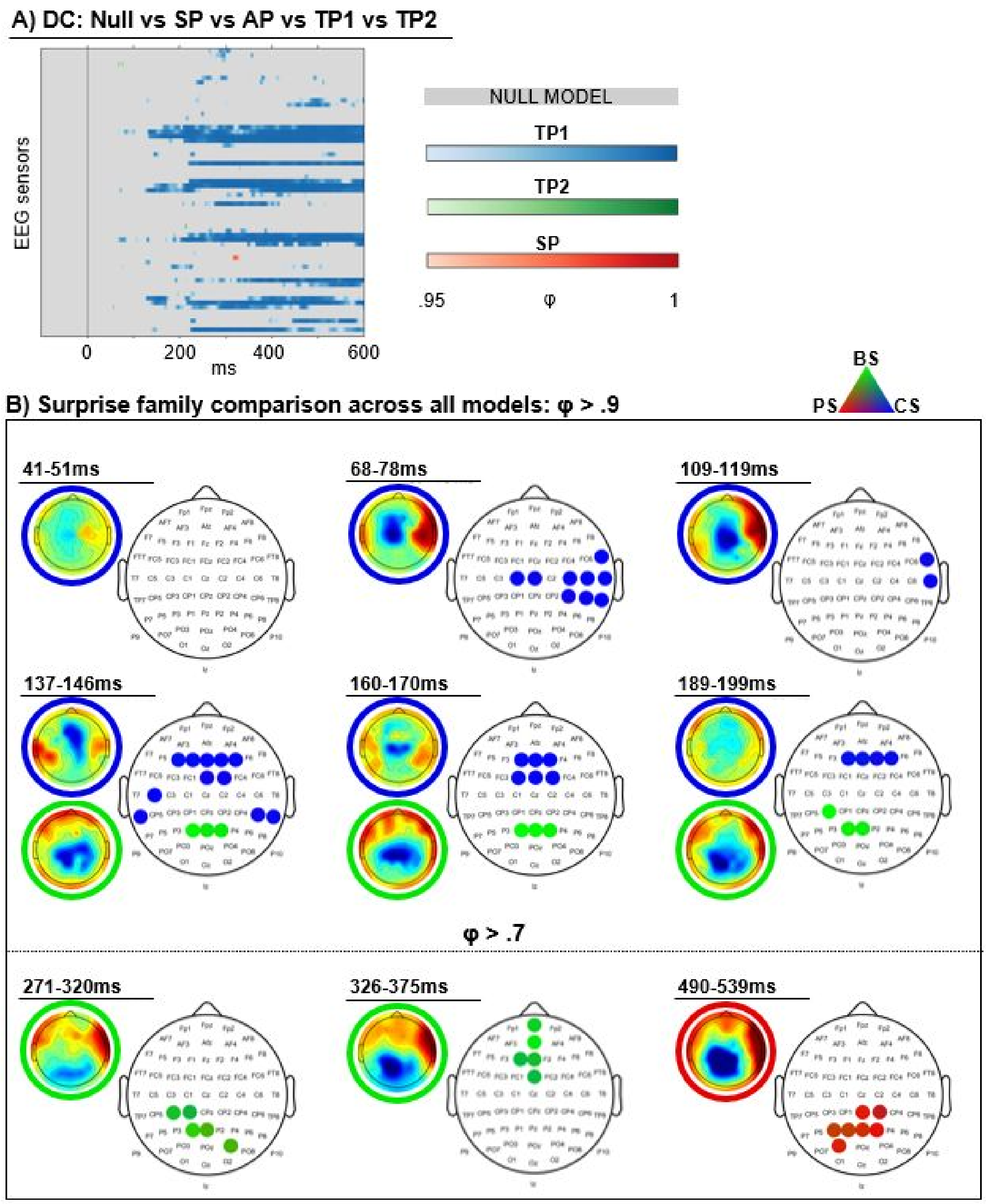

S5 Fig. Non-hierarchical family-wise comparison. Exceedance probabilities (φ) resulting from the RFX family model comparison by investigating the full model space in each comparison. A) Family comparison of the first order transition probability (TP1), second order transition probability (TP2) alternation probability (AP; not shown as no electrode and time-point with φ>0.95) and stimulus probability (SP) models; thresholded at φ>0.95. B) Family comparison of surprise models, thresholded at φ >0.9 (prior to 200ms) and φ>0.7 (post 200ms): Large discrete topographies show the significant electrode clusters of predictive surprise (PS) in red, Bayesian surprise

(BS) in green and confidence-corrected surprise (CS) in blue. Small continuous topographies display the converged variational expectation parameter (m_β).

Addition to the text of the revised manuscript to report on non-hierarchical model comparison (Results l. 591):

We note that the DC TP1 vs TP2 comparison in Fig 10. subplot B has few significant results prior to 200ms. This appears to fit with the model recovery study indicating that the least recoverable families are DC TP1 and TP2 in case of CS and the observation that CS is a winning surprise model for early time bins. In response, we conducted an additional family comparison between SP, AP, and TP encompassing both TP1 and TP2 (see supplementary figure S7 Fig). Clearly, more significant early results can be observed, suggesting that early effects are driven by TP inference but that for empirical data, we are unable to convincingly resolve TP1 and TP2 for CS computation. Although exceedance probabilities have been shown to be inflated (Rigoux et al., 2014), we here opt to report them to allow for a consistent reporting of the same statistic across all levels. Nevertheless, we additionally show the protected exceedance probabilities where possible (as they are unavailable for family comparisons), and expected posterior probabilities otherwise, in S8 Fig. Despite these statistics being diminished, they yield highly similar conclusions, suggesting the results are not solely due to exceedance probability inflation. **In a further control analysis, we performed non-hierarchical model comparison. This procedure grouped the entire model space in the respective families of interest without step-wise data reduction and broadly replicates the findings from the hierarchical approach across the levels (S5 Fig.).**

Exceedance probabilities: You use exceedance probabilities for all comparisons. These are known to be inflated and should whenever possible be replaced by protected exceedance probabilities (Rigoux et al, Neuroimage, 2014, !). I think it would be good if you showed plots of the expected probabilities for all comparisons if you cannot use protected exceedance probabilities which unfortunately are not available for family comparisons. Seeing the expected probabilities will give the reader an idea of the probabilities of individual models and families.

We thank the reviewer for the comment. As mentioned, since protected exceedance probabilities (EPs) are not available for family comparisons we intended a consistent presentation of the results using the same statistic (EPs) across all steps of the hierarchical model comparison. However, we do also appreciate the reviewer's interest in alternative statistics such as the expected posterior probabilities and protected EPs which we are now providing in the supplementary material (S8 Fig.). As pointed out by the reviewer, the protected EPs for the surprise comparison are lower due to their robustness against inflation. The pattern of the results prior to 200ms does not change and the electrode topography shows the same clusters of winning models as reported in the manuscript with protected EPs ($\tilde{\varphi}$) above 0.8. After 200ms the surprise models are less clearly distinguished which becomes apparent by protected EPs of above 0.5.

Furthermore, we noticed that we failed to report this reduced model differentiability in the later time window for the manuscript results. Specifically, at a threshold of φ>0.9, no clusters are found after 200ms, and thus a lower threshold of φ>0.7 was applied for the late clusters in the manuscript, which was erroneously reported as φ>0.9. We are grateful to the reviewer for his request allowing us to detect this unfortunate error. We have adjusted its reporting in figure (Fig. 10) and the results text accordingly.

Changes in the revised manuscript:

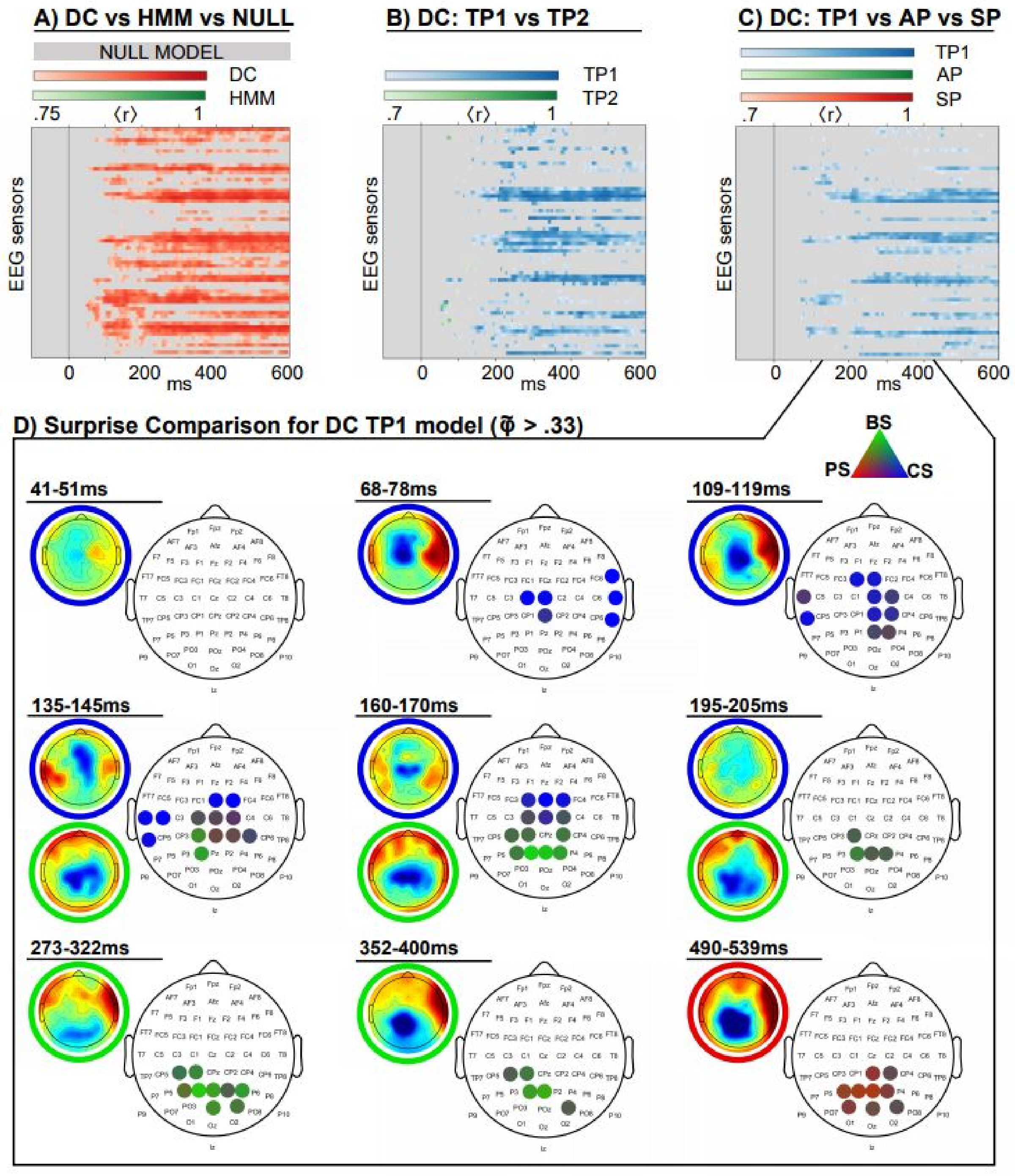

S8 Fig. Alternative statistics of modeling results: Expected posterior probabilities ([r]) and protected exceedance probabilities ($\tilde{\varphi}$). A) Dirichlet-Categorical (DC) model, Hidden Markov Model (HMM) and Null model family comparison, thresholded at [r]>0.75. B) Family comparison within the winning DC family, thresholded at [r]>0.7: first and second order transition probability models (TP1, TP2). C) Family comparison

within the winning DC family, thresholded at [r]>0.7: first order transition probability (TP1), alternation probability (AP) and stimulus probability (SP) models. D) Family

comparison of surprise models within the winning DC TP1 family, without threshold (depicted by $\tilde{\varphi}$>0.33): Large discrete topographies show the electrode clusters of Predictive surprise (PS) in red, Bayesian surprise (BS) in green and confidence-corrected surprise (CS) in blue. Small continuous topographies display the converged variational expectation parameter (m_β).

Methods edit to correct reporting of threshold (l. 435):

The furnished model evidences were subsequently used for a random-effects analysis as implemented in SPM12 [61] to determine the models' relative performance in explaining the EEG data. In order to combat the phenomenon of model-dilution [65], a hierarchical approach to family model comparison was applied (for a graphical overview see S4 Fig). Note that this procedure is performed for each peri-stimulus time bin independently. In a first step, the two model classes DC and HMM were compared against each other and the null-model in a family-wise BMS. A threshold of exceedance probabilities φ>0.99 in favour of either the DC or HMM was applied, so that only whenever there was very strong evidence in favour of one of the model classes the following analyses were applied. For timepoints with exceedance probabilities above this threshold, a family-wise comparison of TP1 and TP2 was performed in order to determine which order of transition probabilities would be used for the second level. Subsequently, either the TP1 or TP2 models were compared to the SP and AP models. Wherever φ>0.95 for one of the inference type families, the third analysis level was called upon. On this final level, surprise read-out functions were compared for the winning model class and corresponding inference type with a threshold of φ>0.9. **As no significant effects were observed after 200ms, the threshold was lowered to φ>0.7 for that time window only.** As such, this step-wise procedure allows spatio-temporal inference of the read-out functions for which there is strong evidence of the belonging model class and inference type. The same procedure was used for the EEG sensor and source data.

Addition to the text of the revised manuscript to report on the alternative-statistic analysis (Results l. 585)

We note that the DC TP1 vs TP2 comparison in Fig 10B has few significant results prior to 200ms. This appears to fit with the model recovery study indicating that the least recoverable families are DC TP1 and TP2 in case of CS and the observation that CS is a winning surprise model for early time bins. In response, we conducted an additional family comparison between SP, AP, and TP encompassing both TP1 and TP2 (see supplementary figure S7). Clearly more significant early results can be observed, suggesting that early effects are driven by TP inference but that for empirical data, we are unable to convincingly resolve TP1 and TP2 for CS computation. **Although exceedance probabilities have been shown to be inflated (Rigoux et al., 2014), we here opt to report them to allow for a consistent reporting of the same statistic across all levels. Nevertheless, we additionally show the protected exceedance probabilities where possible (as they are unavailable for family comparisons), and expected posterior probabilities otherwise, in S8 Fig. Despite these statistics being diminished, they yield highly similar conclusions, suggesting the results are not solely due to exceedance probability inflation.** In a further control analysis, we performed non-hierarchical model comparison. This procedure grouped the entire model space in the respective families of interest without

step-wise data reduction and broadly replicates the findings from the hierarchical approach across the levels (S5 Fig.).

Fitting of tau and model evidence correction: In order to correct for the fitting of tau (the forgetting in the DC models), you subtract "the degree to which tau optimization on average inflated model evidences". First, I do not fully understand the procedure. Average over subjects, over voxel-timepoints? Second, I am not sure this heuristic properly accounts for the additional complexity introduced by tau. Do you have a reference that shows that this heuristic properly controls for complexity? You might be correcting too little or even too much, in which case, your results would become even clearer. In favor of your selection of DC as the winning model class you state in the discussion that the HMM did never win, when tau=0. Does that mean that the DC still clearly won in all these cases? I think you should show the same map as in Figure 12A also for the case of tau=0. This would help to understand the impact of fitting tau. Ideally, the fitting of tau (including defining a prior) should be part of the model inversion, but this might be a larger effort going beyond the scope of this paper. However, I think you should mention this option in the discussion.

This section indeed benefits from a more detailed account of the procedure and we would like to thank the reviewer for this suggestion. As such, we have included this in the main text (please see the correction below). We also now provide the same plot as Figure 10A also for the case of tau=0 as part of S7 Fig which we reproduce below. We acknowledge that the penalization is heuristic and not based on literature reference. However, the case of tau=0 without penalization and the fitting of tau with penalization leads to similar results, with potentially a minor reduction in significant results due to the penalization. This possibly conservative approach was considered to suffice for the scope of this experimental work. Nevertheless, we now make the option of including the fitting of tau in the model inversion explicit to the reader.

Changes in the revised manuscript (l. 411):

Before modeling single subject, single peri-stimulus time bin data (y) as described above, the single-trial regressors of all non-null models as well as the data underwent z-score normalization to allow for the use of the same model estimation procedure for both sensor and source data. For single subjects, data and regressors corresponding to the five experimental runs were concatenated prior to fitting. To allow for the possibility that the brain estimates statistics computed across multiple timescales of integration [9, 63, 64], the forgetting-parameter of the DC model was optimized for each subject, model, and peri-stimulus time-bin. To this end, DC model regressors were fitted for a logarithmically spaced vector of 101-values on the interval of 0 to 1 and the value of tau that resulted in the highest model evidence was chosen. To penalize the DC model for having one of its parameters optimized, the degree to which tau optimization on average inflated model evidences was subtracted prior to the BMS procedure. **Specifically, the difference in model evidence between its average for all parameter-values and the optimized value was computed and subsequently averaged across post-stimulus timebins, sensors, and subjects. The optimization of the forgetting parameter may also have been included in the model inversion itself, although this extends beyond the scope of the current work.**

Reproduction of the new supplementary figure S7A Fig as provided above. This subplot shows that the DC model without forgetting (i.e. tau=0) and no penalization also convincingly wins over the HMM.

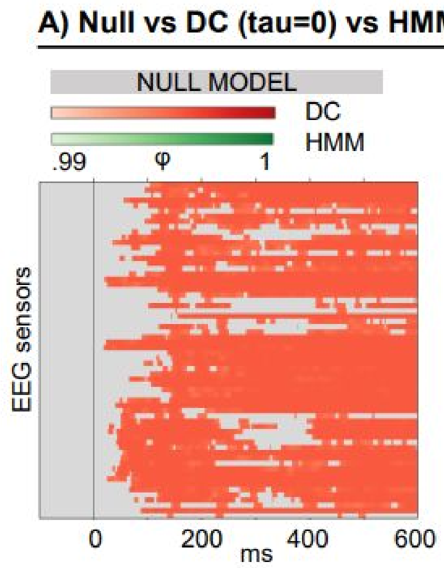

Conclusion of Bayesian learning: You conclude that "early somatosensory cortex seems to reflect Bayesian perceptual learning" (lines 733/734). From your analysis, it is difficult to make a statement about the Bayesian part. All learning models that you tested are Bayesian in nature (except for the null model), hence it could well be that a non-Bayesian model could also provide a good explanation of the data. We simply do not know.

This is a valid point and we thank the reviewer for commenting on it. In order to make a more appropriate claim, we changed the sentence (l. 860) to:

In conclusion, we report evidence that signals of early somatosensory processing can be accounted for by (surprise) signatures of Bayesian perceptual learning.

Inconsistencies in hierarchical scheme: There are a couple of questions to your hierarchical scheme. These are however only relevant, if you would like to stick to it. I just mention them here, and I think you would have to answer them convincingly, if you stick to this scheme.

1.) Why are the thresholds changing for every level?

The thresholds are gradually lowered across the steps of the hierarchical random effects analysis such that the first comparison against the null-model is conservative (strict threshold of $\varphi > 0.99$) in order to avoid carrying false positives over to the subsequent tests. Since multiple comparisons are conducted on the same electrode and time-bin we allowed for lower thresholds in subsequent analysis steps on remaining data that has already been thresholded. We now clarify this in the Methods.

Changes in the revised manuscript (l.433):

On this final level, surprise read-out functions were compared for the winning model class and corresponding inference type with a threshold of $\varphi > 0.9$. **We allowed for lower**

thresholds for these second and third analysis steps on remaining data given a threshold had already been applied.

2.) What is the rational for splitting up the comparison over TP1, TP2, SP and AP into two? I think this should be one single model comparison. (In fact, this leads to a misinterpretation of results when you say "Our results show that the TP model family clearly outperformed the SP and AP families." What you show is that TP1 outperforms these other families.) Why are you reevaluating in places/at timepoints where TP1 does not win? This deviates from the general strategy.

Given that SP, AP, and TP are different sequence statistics while TP1 and TP2 refer to different orders of the same sort of statistic this was considered to be a conceptually sound distinction, in addition to consequent concerns over possible model dilution between the TP variants in case of an SP, AP, TP1, TP2 comparison. The addition of the model recovery study sheds light on this issue, as it appears to be specifically for the case of confidence corrected surprise that TP1 and TP2 are difficult to recover. This can be seen in the experimental results in Fig 10B as well, with only few significant results prior to 200ms, which is precisely where we end up finding CS as the winning surprise model (a finding that is replicated in the non-hierarchical model comparison). We concluded that a comparison of TP1 and TP2 should precede the comparison with SP and AP in order to include only the overall more likely TP model in the hierarchical model comparison. Likewise, TP2 was not used for further thresholding of the surprise results in addition to TP1.

An additional comparison was performed with TP1 and TP2 grouped into one TP family when compared to SP and AP (see supplementary figure S7 Fig. C), which replicates the TP-effects prior to 200ms, indicating a TP-based computation also for CS. Importantly, with the help of the results of the model recovery study, we have edited the text to reflect the lower confidence in the order of TP inference underlying CS computation.

Addition to the text of the revised manuscript to report on TP1 vs TP2 family comparison (Results l. 578)

We note that the DC TP1 vs TP2 comparison in Fig 10. subplot B has few significant results prior to 200ms. This appears to fit with the model recovery study indicating that the least recoverable families are DC TP1 and TP2 in case of CS and the observation that CS is a winning surprise model for early time bins. In response, we conducted an additional family comparison between SP, AP, and TP encompassing both TP1 and TP2 (see supplementary figure S7). Clearly more significant early results can be observed, suggesting that early effects are driven by TP inference but that for empirical data, we are unable to convincingly resolve TP1 and TP2 for CS computation. Although exceedance probabilities have been shown to be inflated (Rigoux et al., 2014), we here opt to report them to allow for a consistent reporting of the same statistic across all levels. Nevertheless, we additionally show the protected exceedance probabilities where possible (as they are unavailable for family comparisons), and expected posterior probabilities otherwise, in S8 Fig. Despite these statistics being diminished, they yield highly similar conclusions, suggesting the results are not solely due to exceedance probability inflation. In a further control analysis, we performed non-hierarchical model comparison. This procedure grouped the entire model space in the respective families of interest without

step-wise data reduction and broadly replicates the findings from the hierarchical approach across the levels (S5 Fig.).

Discussion (l.742):

In order to investigate which statistics are estimated by the brain during the learning of categorical sequential inputs, we compared three models within the DC model family that use different sequence properties to perform inference on future observations: stimulus probability (SP), alternation probability (AP), and transition probability (TP) inference. The TP model subsumes SP and AP models and is thus more general by maintaining a larger hypothesis space. Our results show that the TP model family clearly outperformed the SP and AP families, thereby suggesting that the brain captures sequence dependencies by tracking transitions between types of observations for future inference. We thereby provide further evidence for an implementation of a minimal transition probability model in the brain as recently concluded from the analysis of several perceptual learning studies [90], extending it to include somesthesis. Additionally, we expand upon previous studies by comparing a first order TP model (TP1), capturing transitions between stimuli conditional only on the previous observation, with a second order TP model (TP2), which tracks transitions conditional on the past two observations. Our results suggest that the additional complexity of the second order dependencies contained in our stimulus sequence were not captured by the brain, **although we were not able to convincingly show this for early CS computation.** Nevertheless, the brain may resort to alternative, more compressed representations [91].

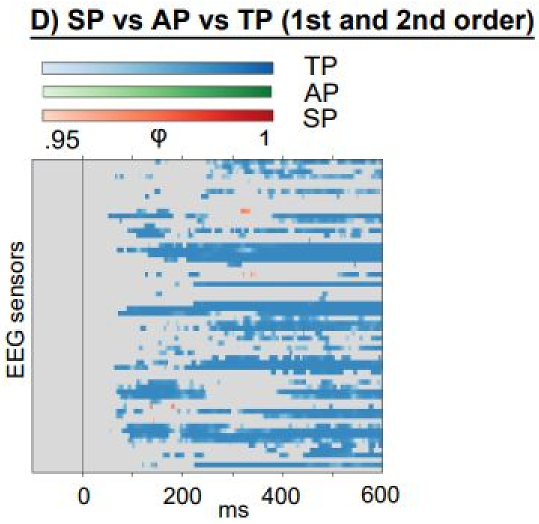

S7 Fig. Additional random effects family comparisons. D) Comparison of the model families within the DC model: Stimulus probability model (SP), alternation probability model (AP) and transition probability model family (TP) subsuming first and second order TP models in one family.

3.) Why did you choose this exact order of hierarchy? What would a different ordering yield?

The hierarchical order of our analysis was intended to develop from general to more specific principles. On the broadest level, our models are two different classes of model architectures for Bayesian inference (HMM and DC models). Each of these model classes may be specified to track different sequence statistics: stimulus, alternation, and transition probabilities. Finally, these statistics may be used to perform surprise computations, which can be interpreted as the readout functions of the models, which are naturally dependent on both the model class as well as which sequence statistic is tracked. Specifically, the model class and sequence statistic determine the probability estimates of the models, while the surprise computation is only a readout of this estimate. As such it appeared to us to be the most conceptually sound ordering that yields interpretable results also at the intermediate levels.

As suggested by the reviewer, we also inspected the non-hierarchical family comparison as a reasonable alternative approach. This procedure groups the entirely model space in the respective families of interest without step-wise data reduction. The results of both approaches are highly similar, suggesting that our results are generally robust against changes in the exact strategy of model comparison.

4.) Even if you stick to the hierarchical scheme, which I do not recommend, I think you would have to show the expected model probabilities for all models and family comparisons. The reader should be able to appreciate that the final decision for a single model, although it might be clear in the final step, is only performed within a probably small fraction of the entire mass of your model space. It is probably not feasible to show this for all voxel-timepoints, but you could select a couple of representative examples.

As described in the reply above we now provide the expected posterior probabilities and protected exceedance probabilities (where they apply) in a supplementary figure (S8 Fig.) in order for the reader to inspect the effects in light of these alternative statistics. As we previously showed that similar conclusions result from a hierarchical and non-hierarchical scheme, we hope this current analysis further justifies the reporting of the results of the hierarchical scheme by showing that they are not significantly dependent on the choice of statistic. With the additional argumentation added to the text as outlined above, we hope to make it clear to the reader that the approach performs model comparison on a fraction of EEG sensors, time points, and models as one moves through its levels.

5.) How can you assure that your statements about the best model hold?

We submitted the model comparison to a simulation study which is described in the reply above as well as in an addition to the methods section of the manuscript . No false positives were observed in the simulation under a relevant signal-to-noise level. Additionally, as mentioned above with regards to the non-hierarchical model comparison, the presented pattern of results does not seem to be dependent on the exact model comparison procedure which is in favour of the claimed statements about the best model.

Minor points:

Multiple comparison for Bayesian Model Selection: Doing model or family comparison for every single voxel-timepoint means that you are conducting many model comparison tests. I am not sure there is a solution for this problem, but it might be worth mentioning this. I do not think this invalidates any findings at particular levels, but it might be good to remind the reader that the voxel-timepoints with preference for a particular family are just few of many that were tested.

We thank the reviewer for their comment on the issue of multiple comparison correction for Bayesian Model Selection and reproduce our reply to reviewer #2:.

In Bayesian model comparison there is no conventional way to correct for multiple comparisons and it has been established that Bayesian methods provide inherent adjustments of sensitivity and specificity to deal with false positive rates (Friston 2002, Neuroimage, doi:10.1006/nimg.2002.109 and Friston 2002, Neuroimage, doi:10.1006/nimg.2002.109). However, in line with a comment of reviewer #3 (below) we added information on the number of comparisons in the text.

Line 189: How was train length entered in the GLM? As a parametric modulator, or as several modulators each coding for one length?

The train length was entered as five modulators each coding for one train length, which we now more clearly document in the manuscript. The inspection of the respective section brought to our attention an unfortunate misreporting in the GLM description. While we write to have included regime in the GLM we report on, we in fact ran a separate GLM in order to test for regime effects. We opted for this approach as balancing the number of standards and deviants between the regimes lead to considerably less total trials. This balanced GLM showed no main effect of regime or interaction of regime and stimulus type. We now accurately report this in the description of the GLM analysis (l.169):

On the first level, the single-trial data of each participant was subject to a multiple regression approach **with several regressors each coding for a level of an experimental variable: stimulus type (levels: standard and deviant), train length (levels: 2, 3, 4, 5, >6 stimuli) and a factor of experimental block as nuisance regressors (levels: block 1-5). An additional GLM with a balanced number of standard and deviant trials for the regimes (levels: fast and slow switching regime) showed no effect of regime or interaction of regime and stimulus type.**

Line 255: I think there is a typo in the right hand side of the equation. One of the j indexing s_t and s_t-1 should be an i.

Thank you for bringing the typo to our attention, we have corrected it and the respective part of the equation in line 255 now reads:

$$p(s_t^j|s_{t-1}^i)$$

Fig. 5: The x-axis label is probably trial number and not time in ms.

We thank the reviewer for pointing out this unfortunate mistake and have changed the x-axis label to trial number.

Fig 8: Reference to panel E is missing in caption.

We thank the reviewer for pointing out the missing reference to panel E in the caption and added it.

Fig 9: Please remind the reader of the coloring of deviants and standards (bottom row). I assume this is the same coloring as in Figure 1.

We thank the reviewer for pointing out the missing legend in Fig. 9. The coloring indeed resembles Fig. 1 and we added the color reference in the caption.

Fig. 10: Are the values for rS2 and lS2 correct. Shouldn't the "Moment Posterior" be symmetric as well?

We thank the reviewer for this observation. Even though we used a symmetric pair for the equivalent current dipole fitting procedure in SPM (VB-ECD), the method uses "soft" symmetry constraints (as described in Fastenrath, Friston & Kiebel 2009, Neuroimage, doi: 10.1016/j.neuroimage.2008.07.041) which, in our case, results in slightly different orientations of left and right S2 dipoles to best account for the data.

Changes in the revised manuscript (l.530):

The distributed source reconstruction resulted in significant clusters at the locations of primary and secondary somatosensory cortex (Figure 8A, with details specified in the corresponding table). The resulting anatomical locations were subsequently used as priors to fit four equivalent current dipoles (Figure 8B, with details specified in corresponding table). Two dipoles were used to model S1 activity at time points around the N20 and the P50 components while an additional symmetric pair captured bilateral S2 activity around the N140 component. **The moment posteriors of the S2 dipoles end up not strictly symmetric due to the soft symmetry constraints used by the SPM procedure (Fastenrath, Friston & Kiebel 2009).**
