# Supplementary material for: Neural surprise in somatosensory Bayesian learning
Source: PLoS Comput Biol. 2021 Feb 2;17(2):e1008068. doi: 10.1371/journal.pcbi.1008068 (PMC7880500; doi:10.1371/journal.pcbi.1008068)
Supplement: Attachment — Submitted filename: ResponseToReviewers_PCOMPBIOL-D-20-01012R2.pdf [file pcbi.1008068.s011.pdf]

Prof. Dr. Samuel Gershman
Deputy Editor
PLoS Computational Biology

Dr. Philipp Schwartenbeck
Guest Editor
PLoS Computational Biology

Revised manuscript: PCOMPBIOL-D-20-01012R1, “Neural surprise in somatosensory Bayesian learning”

Dear Prof. Dr. Samuel Gershman, dear Dr. Philipp Schwartenbeck,

As encouraged by your letter of October 16, please find attached the revision of our manuscript (PCOMPBIOL-D-20-01012R2).

We are very grateful for the extensive effort taken by the three reviewers to consider our resubmission and their continued support in improving the submission by providing helpful insight and important comments. We were pleased to read that the reviewers consider the manuscript a valuable contribution and we believe their relevant suggestions have led to a considerably improved revision.

In brief, the notable additions include a critical discussion of the model comparison scheme and the acknowledgement of its potential limitations. This encompasses a clarification of our perspective on the relationship between the multiple testing problem and the large number of Bayesian model selections reported in the current manuscript. We aimed to address the concerns regarding the reporting of the modeling results by adapting the expressed confidence of the various effects appropriately and implementing changes to the thresholds and reported statistics. Furthermore, we performed additional analyses to inspect the model-derived predictions for the ERP contrasts. We now include a section that discusses these predictions and the extent to which they fit the observed EEG results. Please see our response to the reviewers for details of each of the applied changes.

We thank you and the reviewers for your consideration and helpful comments, and hope that you will find our study to be suitable for publication in *PLoS Computational Biology.*

We look forward to your reply.

Sincerely,

Sam Gijsen, Miro Grundei, Robert T. Lange, Dirk Ostwald, Felix Blankenburg

Reviewer #1:

Thank you for asking me to re-review this manuscript. I would like to thank the authors for engaging with my comments, which primarily concerned the interpretation of the findings. I am satisfied both by their responses and additions to the Discussion section. I believe the manuscript has been greatly improved.

We thank the reviewer again for their valuable input to improve the manuscript.

Reviewer #2:

The authors have put some work in addressing my concerns. I particularly found the new Figure 5 very insightful. However, I am not fully convinced by all of their responses. In particular, this concerns:

a) the relationship between the conventional ERP analysis and the model-based single-trial analysis

b) their statistical thresholds and analysis choices.

These points would need to be addressed before I could support publication.

Sincerely,

Lilian Weber

Comments to the authors:

Thank you for comprehensive replies to my concerns and the considerable effort you put into this revision. I particularly appreciated Figure 5, and some of the clarifications you provided in your responses regarding your overall hypotheses and the scope of your approach.

However, I was not convinced by some of your replies. I list these below with comments.

a) conventional ERP results and single-trial model-based analysis

In my previous comment, I wrote:

"I would encourage the authors to address this question, for example by deriving predictions from the different models for MMR effects: (how) does the MMR arise from differences in surprise between trials labeled as standards and those labeled as deviants in the conventional analysis? What predictions do the models make about the effects of train length on surprise? Is the winning model compatible with the experimental observations for the different MMRs?"

I appreciated your addition to the Discussion about the potential relationship between ERP components and the EEG correlates of your surprise measures, which I found offered a very sensible interpretation. However, I still think you could make much more specific statements by looking at what the different models would predict in terms of a standards vs deviants contrast. Importantly, this to me does not seem to require a disproportionate effort: you only have to apply the conventional trial definition to your model-based surprise readouts. After all, you motivate your study with the question of which mechanisms underlie the classically observed mismatch signals (l.35-38).

Figure 5 was already helpful in understanding the specific predictions that the different models make for single-trial ERP responses, which could explain their differential performance in predicting the EEG amplitudes. For example, CS in the DC model predicts these slow drifts within trains of stimuli, where variance (between standards and deviants) decreases, but the overall mean surprise increases. Using these model-based single-trial surprise measures you can easily derive MMR predictions by averaging surprise to standards versus deviants.

For example, this could illustrate your point: "This counteracting effect of belief commitment and the surprise terms can lead to independence of CS and train length when responses are averaged", or you could show which surprise measures would predict the lack of difference between MMRs in the stable versus the volatile regime.

The traditional definition of standards and deviants is a heuristic for what is surprising or predictable, and implicitly suggests a model of how observers perceive the sequence: e.g., repetitions are more likely than transitions. Or: observers track the stimulus probability for every trial, and settle on a higher probability of the repeated stimulus towards the end of a train, then update at the onset of the new train. Your single-trial analysis uses more information (i.e., all trials) from the data to make a more precise statement about underlying mechanisms, but from your current results it remains unclear if the classically observed MMRs correspond at all to differences in surprise between standard and deviant trials as quantified by your winning model.

We thank the reviewer for these comments. To address the reviewer's points, we analysed the parametric effect of the preceding standard sequence length on surprise. The suggested analysis shows that the three MMRs found in the ERP analyses and their relation to train length are differentially supported by the model-derived predictions. We expand the relevant paragraph in the discussion to comment on these results. The model predictions are dependent on the forgetting-parameter tau, which led us to investigate the predictions made by a few diverse parameter values (ranging from no forgetting to very high forgetting). In the discussion section we focus mainly on those models and parameters that were found to best fit the single-trial data (DC TP1 BS with half-life=26 and DC TP1 CS with representative half-life=95). The relevant figure showing the averaged surprise readouts is included as a Supplementary Figure (S4 Fig.) for the revised manuscript.

Changes in revised manuscript (Methods l. 379):

In an exploratory analysis, the trial-definitions of the GLM analysis of the individual electrode-time point data were applied to the surprise readout regressors. This allowed for the derivation of model-based predictions for the observed beta-weight dynamics of the ERP GLM. First, we generated an additional 25000 sequences of 800 observations using the same generative model used for the subject-specific sequences. The averaged surprise readouts of these simulated sequences yielded model-derived predictions, which allowed for a visual verification of the presence of these predictions in the (200) experimental sequences. As each study subject was exposed to 5 sequences, these sequences were grouped into sets of 5 (yielding 5000 simulated subjects) to mirror the EEG analysis. Besides the HMM, we used the Dirichlet-Categorical models with different values for the forgetting-parameter ('no forgetting' and long, medium-length, and very short stimulus half-lives) (S4 Fig.). To reduce the model-space, only TP1 models were used for this analysis.

Discussion (l. 834):

In an attempt to relate the surprise readouts to the mismatch responses, we averaged surprise regressors to obtain model-based predictions for the standard-deviant

contrasts. First, all TP1 models except HMM CS predict the existence of an MMR, i.e., a difference in the averaged response between standard and deviant trials. Second, for multiple models an increase in train length leads to reduced surprise to standards and increased surprise to deviants. The CS readout is scaled by PS and BS, as well as by belief commitment, which increases for standards and decreases for deviants. This counteracting effect of belief commitment and the surprise terms can lead to independence of CS and train length when responses are averaged, manifesting in the current sequences only for standard trials. As the early MMR was found to be independent of train length, this indicates a possibility for a potential relation between these results. The intermediate MMR roughly temporally co-occurs with a simultaneous representation of BS and CS in S1 and S2. The dependence of the mid-latency MMR on train-length for both standards and deviants and the encoding of belief inadequacy and updating quantities is suggestive of convergent support in favor of a perceptual learning response which involves both somatosensory cortices. DC BS is however not the only model which predicts this dependence, highlighting the reduced ability to distinguish between models by averaging trials. At the P300 MMR it was found that only the response to deviants is dependent on train length. The averaged response of DC CS is most compatible with this ERP, however, this is unlikely to be meaningful as the model was not found to fit the single-trial EEG data well around this time. It is noteworthy that belief updating as described by DC BS, which is best describing the EEG data around that time, does not accurately predict the ERP dynamics of the P300, which matches the relative weakness of the BS effect in the single-trial EEG analysis. While a role of the P300 response in Bayesian updating has previously been reported [13, 40], the currently presented P300 dynamics may better be captured by alternate accounts, such as a reflection of an updating process of the attention allocating mechanism as suggested by Kopp and Lange [106].

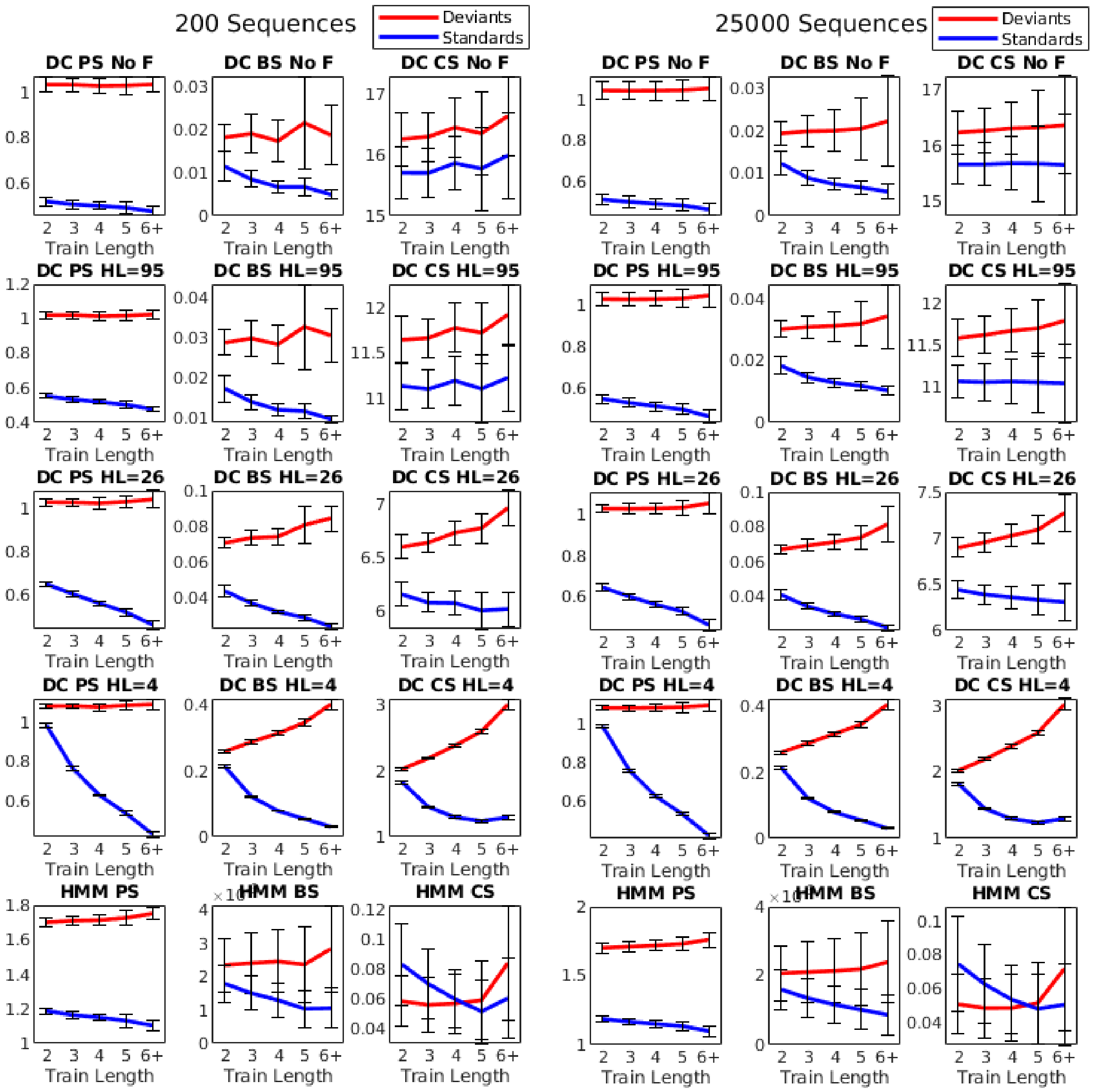

S4 Fig.: Averaged surprise readouts using either the (left) 200 sequences administered to the participants or (right) 25000 total sequences elicited for standard and deviant stimuli following a certain amount of repeating stimuli (train length). The model-derived predictions are relatively well-preserved in the smaller data-set. Only first-order transition probability models are plotted. Error bars indicate standard deviations. DC: Dirichlet-Categorical model; HMM: Hidden Markov Model; PS: Predictive surprise; BS: Bayesian surprise; CS: Confidence-corrected surprise; No F: model without forgetting (i.e. perfect integration); HL: stimulus half-life.

b) analysis choices

- multiple comparisons:

You write: "In Bayesian model comparison there is no conventional way to correct for multiple comparisons and it has been established that Bayesian methods provide inherent

adjustments of sensitivity and specificity to deal with false positive rates (Friston 2002, Neuroimage, doi:10.1006/nimg.2002.109 and Friston 2002, Neuroimage, doi:10.1006/nimg.2002.109)."

I cannot agree with this point and I don't see how the cited papers relate to your analysis. As far as I can see, these deal with hierarchical Bayesian models (parametric empirical Bayes), whereas you perform separate, independent model comparisons per voxel (sensor and time point). I'm happy to be corrected here.

We thank the reviewer for pointing out the erroneous implication that the inherent adjustments in the hierarchical Bayesian models discussed in the citations are of relevance for our analysis scheme. A similar concern was raised by reviewer #3 and we acknowledge that we do not provide a corrective measure for the large amount of independent model comparisons that are performed. However, these model comparisons do not constitute statistical tests per se, as they do not provide a mapping from the data to binary outcomes. Rather, the resulting exceedance probabilities are a measure of effect size, which are reported here only above a given threshold. It follows that the analyses do not suffer from a classical multiple testing problem, which can be addressed using the control of multiple testing error rates (e.g. the control of the family-wise error rate for fMRI inference based on random field theory). Nevertheless, we think it would be very valuable for methodological advances to consider the possibility of randomly occurring high effect sizes given a large number of independent model comparisons. As this is currently not methodologically accounted for we here present only preliminary evidence and make this now explicit in the revised manuscript.

<u>Changes in the revised manuscript (Discussion, l. 910):</u>

The analyses performed here include a large number of independent Bayesian model comparisons (as is not uncommon in neuroimaging), yet no corrections are applied. These model comparisons do not constitute statistical tests per se, as they do not provide a mapping from the data to binary outcomes. Rather, the resulting exceedance probabilities are a measure of effect size, which are reported here only above a given threshold. It follows that the analyses do not suffer from classical multiple testing problem, which can be addressed using the control of multiple testing error rates (e.g. the control of the family-wise error rate for fMRI inference based on random field theory). Nevertheless, it would be valuable for methodological advances to consider the possibility of randomly occurring high effect sizes given a large number of independent model comparisons. A multilevel scheme which adjusts priors over models, rather than the current ubiquitous use of flat priors, may be developed as a satisfactory approach (Friston et al., 2002; Gelman et al., 2012; Neath, et al., 2018). As the current method is agnostic to the large number of model comparisons we need to stress that we only report preliminary evidence.

- catch trials:

I appreciate your reanalysis and including it as a supplementary figure. It does seem to me that when excluding catch trials for the DC model, the evidence for the DC model compared to the HMM is significantly reduced (Fig. S7B vs S7A). Given that the comparison without the catch trials is the fairer one, I think this deserves mentioning in the main text.

We thank the reviewer for this suggestion. In order to bring the reader's attention to the reduced evidence for the DC model over the HMM if the DC does not model the catch trials, we have included a statement on this additional comparison in the main text of the manuscript.

Changes in revised manuscript:

Results (l. 584):

For large time windows at almost all electrodes there is strong evidence in favor of the DC model class (φ > 0.99), while the HMM model class does not exceed thresholding anywhere, therefore excluding HMM models from further analyses. To verify that this result was not merely due to an insufficient penalization of the DC models, the analysis was repeated with τ=0. Thus, under this setting, all instances of the DC model had perfect, global integration similar to the HMM models. Likewise, no results above the threshold were found for the HMM model class (S7A Fig). **Next, to ensure that the superiority of the DC model did not solely result from the additionally modeled catch trials, the HMM was compared with a DC model which did not capture these trials. This DC model still consistently outperformed the HMM, though it should be noted that the evidence for such a reduced DC model over the HMM is less pronounced (S7B Fig.).**

Discussion (l. 736):

Our comparison of these two learning approaches provides evidence for the DC model class over the HMM for the large majority of electrodes and post-stimulus time. The superiority of the DC model was found to be irrespective of the inclusion of leaky integration to the DC model, indicating the advantage of a non-hierarchical model in explaining the EEG data. **It is noteworthy that part of the strength of the DC model depended on the modelling of the catch trial, although a reduced DC model still outperformed the HMM.**

- exceedance probability thresholds in the step-wise comparison approach

The different model comparisons (DC vs HMM, TP1 vs TP2, etc.) seem to be orthogonal to each other. Therefore, I don't understand why the thresholds should decrease over the successive steps. The fact that the voxels have been thresholded before does not seem relevant if the comparisons are orthogonal?

We thank the reviewer for their comment and we have adjusted the thresholding in the revised manuscript. As mentioned in reply to a previous comment, the performed Bayesian model comparisons do not constitute statistical tests per se and by extension it was unfortunate that the previous version of the manuscript referred to statistical significance of results. However, as thresholding does not serve to indicate statistical significance we now make explicit that the thresholds are an arbitrary choice to draw conclusions on (and visualize) the effects with a certain minimum effect size. As the main concern seems to be addressing thresholding that is potentially too loose, we decided to stick with a high threshold of EP>0.99 for the first model comparison of [NULL vs DC vs HMM] to be conservative against the null-model. For the subsequent comparisons of [TP1 vs TP2] and [SP vs AP vs TP1] we threshold at 0.95. The final surprise comparison depicted as electrode topographies are provided with demarcations of electrodes that survive a thresholding of protected exceedance probabilities above 0.95. The topographies are otherwise shown without thresholding in order to indicate tendencies of the surprise readouts. The visualization of the dipole-level BMS accordingly now also features protected exceedance probabilities with a visual threshold of 0.95.

Changes in the revised manuscript:

Methods (l. 438):

A threshold of exceedance probabilities φ> 0.99 in favour of either the DC or HMM was applied**, so that only whenever there was strong evidence in favour of one of the**

model classes over both the alternative and the null-model the following analyses were applied.

Methods (Bayesian model selection, l. 440):

As the current analyses are not statistical tests per se, the thresholding of the data by certain exceedance probabilities ultimately constituted an arbitrary choice to reduce data in order to visualize (and draw conclusions on) effects with certain minimum effect sizes within a large model space.

Similarly, further references to the thresholds have been adjusted to reflect the new thresholding in lines 492 and 604.

Results (Modeling in sensor space; Fig. 10):

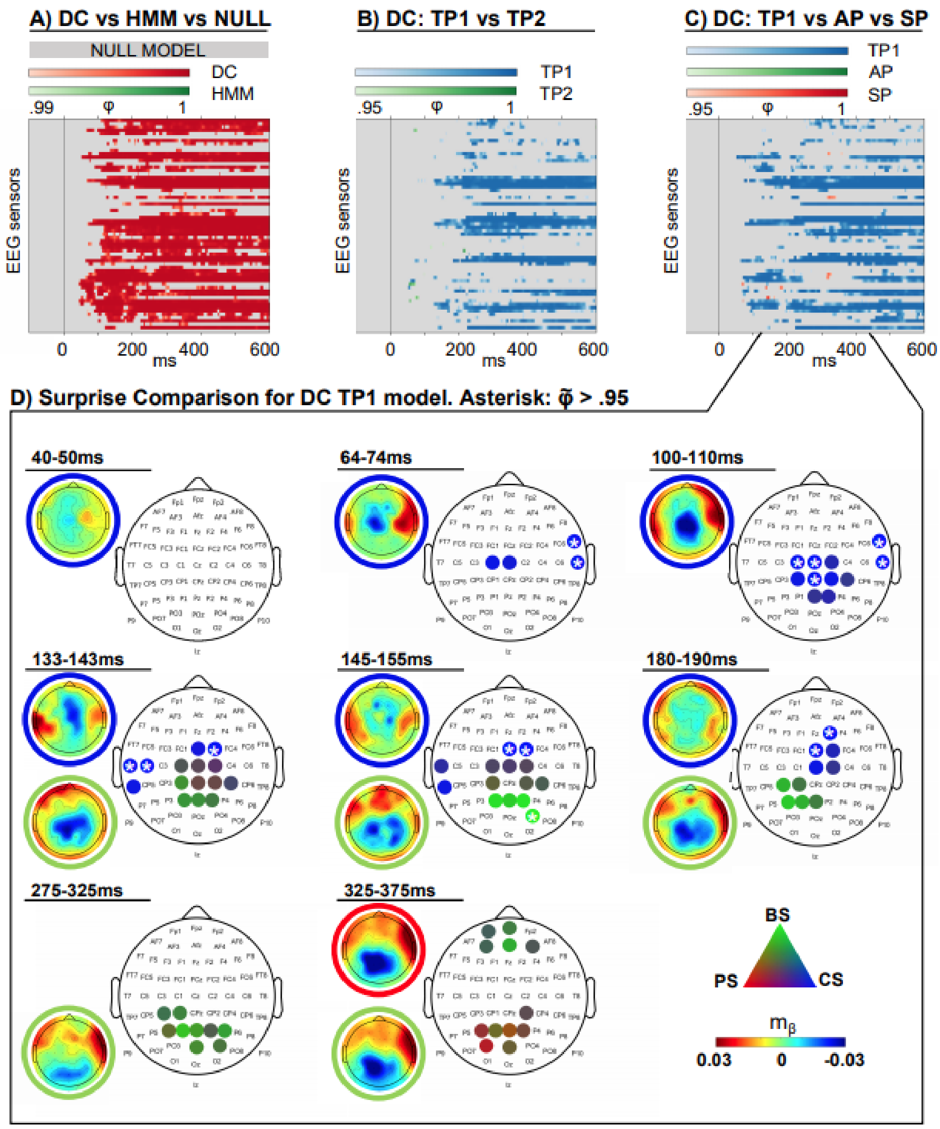

Fig. 10: Exceedance probabilities (φ) resulting from the random-effects family-wise model comparison. (A) Dirichlet-Categorical (DC) model, Hidden Markov Model (HMM) and null model family comparison, thresholded at φ > 0.99 and applied for data reduction at all further levels. (B) Family comparison within the winning DC family, thresholded at φ > 0.95: first and second order transition probability models (TP1, TP2). (C) Family comparison within the winning DC family, thresholded at φ > 0.95: first order transition probability (TP1), alternation probability (AP) and stimulus probability (SP) models and applied at the final level. **(D) Unthresholded protected**

exceedance probabilities ($\tilde{\varphi}$) resulting from model comparison of surprise models within the winning DC TP1 family: Large discrete topographies show the electrode clusters of predictive surprise (PS) in red, Bayesian surprise (BS) in green and confidence-corrected surprise (CS) in blue. White asterisks indicate $\tilde{\varphi}$>0.95 of single electrodes. Small continuous topographies display the converged variational expectation parameter mβ. This parameter may be interpreted as a β weight in regression, indicating the strength and directionality of the weight on the model regressor that maximizes the regressor's fit to the EEG data (see S2 Appendix).

Results (Modeling in source space; Fig. 11):

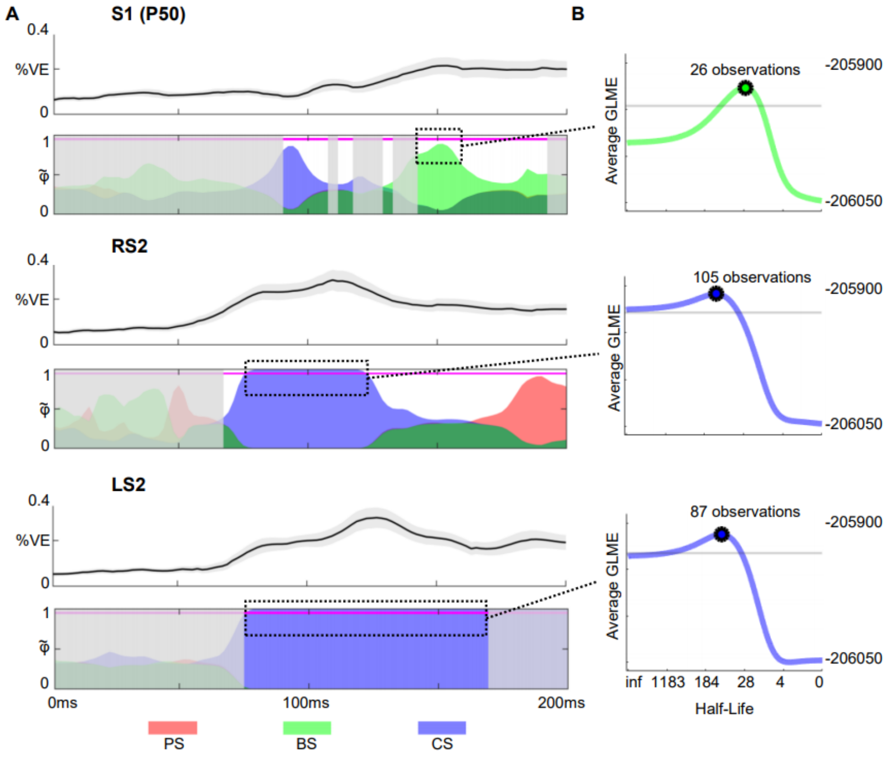

Fig. 11: Modeling results in source space with best fitting forgetting-parameter values. Red: Predictive surprise (PS), Green: Bayesian surprise (BS), Blue: Confidence-corrected surprise (CS) A) **Colored areas depict protected exceedance probabilities ($\tilde{\varphi}$) of the surprise readout functions of the Dirichlet-Categorical TP1 model within the dipoles S1P50, right S2 (RS2) and left S2 (LS2) using alpha blending. In grey shaded areas the DC model family shows φ<0.99 or the TP1 model family φ<0.95. The S1N20 dipole was omitted in the visualization as no model is observed above this threshold. Magenta horizontal lines indicate $\tilde{\varphi}$=0.95. Line plots above each dipole plot show the respective mean percent variance explained of the models in dotted rectangles +- standard error.** B) The group log model evidence (GLME) values corresponding to the stimulus half-lives for forgetting-parameter τ, after averaging the timebins inside the dotted-rectangles **(S1P50: 143-157ms; RS2: 75-120ms; LS2: 75-166ms)**. The grey lines indicate a difference of 20 GLME from the peak, indicating very strong evidence in favour of the peak half-life value compared to values below this threshold.

- comparisons between different surprise readouts

Judging from Fig. 5, I would expect the different surprise measures to be hardly distinguishable, especially BS and PS in the DC model. Indeed, when using protected exceedance probabilities, the evidence for one surprise measure over another seems to be weak at best (Fig. S8). Given that the 'alternative statistics' (expected probabilities and protected exceedance probabilities) are actually the more robust statistics, the data do not seem to provide strong support in favour of one or another surprise measure. (The fact that the results with a lower threshold, i.e., the exceedance probabilities, look similar to the ones with a higher significance threshold does not mean that the exceedance probabilities are not

inflated.) I believe it would be appropriate to tone down your conclusions about different surprise measures reflected in your data.

We agree that some of the presented surprise effects are weak and we have in general toned down the discussion appropriately. We also replaced the exceedance probabilities of the surprise readout models in the main manuscript by the *protected* exceedance probabilities previously reported in the Supplementary Material (see Fig. 10 above). This statistic however does provide relatively strong evidence for the early CS effects, which was not very well conveyed in our earlier manuscript as the colorbar scaling does not resolve the peak probabilities well which we now account for by demarcation of a $\tilde{\varphi}$>0.95 threshold. We now comment on the difference in support between the reported surprise effects accordingly. The major changes concern the following paragraphs:

Results section - Modeling in sensor space (l. 592):

Thus, the following section presents the random-effects Bayesian model selection results of the readout functions of the Dirichlet-Categorical TP1 model (shown in Fig 10D). The scalp topographies depict the winning readout functions of the DC TP1 model at different time windows. Given the difference in temporal dynamics of faster, early (<200 ms) and slower, late (e.g. P300) EEG components, different time windows were applied for averaging. Early clusters were identified by averaging protected exceedance probabilities over 10ms windows and using a minimum cluster size of two electrodes, while 50ms time windows were applied for averaging across later time windows with a minimum cluster size of four. The resulting clusters indicate that from around 70ms on, early surprise effects represented by confidence corrected surprise (CS) best explain the EEG data on contralateral and subsequently ipsilateral electrodes up to around 200ms. As demarcated in the plot, the early CS clusters include electrodes with $\tilde{\varphi}$>0.95, which is indicative of a strong effect size. A weaker cluster of Bayesian surprise (BS) is apparent at centro-posterior electrodes between 140-200ms of which the peak electrodes around 150ms show $\tilde{\varphi}$ between 0.8 and 0.95. As such, the mid-latency BS effect is less strong than the earlier CS clusters and can only provide indications. At the time windows of the P300 around 300 and 350ms, similar centro-posterior electrodes represent weak Bayesian surprise (peak $\tilde{\varphi}$ around 0.75) and predictive surprise (PS) clusters (peak $\tilde{\varphi}$ around 0.72), respectively. The mid-latency BS cluster is temporally in accordance with the putative N140 MMR while the late two clusters of BS and PS might be interpreted as indicative of a P300 MMR. However, especially the weak late clusters do not provide clear evidence in favour of a specific surprise readout function.

Discussion:

l. 662:

Using computational modelling, EEG signals were best described by a non-hierarchical Bayesian learner performing transition probability inference. **Furthermore, we provide evidence for an early representation of confidence-corrected surprise localized to bilateral S2 and weak indications for subsequent Bayesian surprise encoding in S1.** These computations were shown to use a local, rather than global, timescale of integration.

l. 821:

The BS effect around 140ms was less pronounced in source space only peaking at $\tilde{\varphi}$ of 0.89 and was localized to S1. Despite the weak evidence for this BS representation

around a 140ms somatosensory MMR, its timing matches prior work using Bayesian modeling of surprise signals in the somatosensory system [13].

l. 929:

In conclusion, we **show** that signals of early somatosensory processing can be accounted for by (surprise) signatures of Bayesian perceptual learning. The system appears to capture a changing environment using a static latent state model that integrates evidence on a local, rather than global, timescale and estimates transition probabilities of observations using first order dependencies. **In turn, we provide evidence that the estimated statistics are used to compute a variety of surprise signatures in response to new observations, including both puzzlement surprise scaled by confidence (CS) in secondary somatosensory cortex and weak indications for enlightenment surprise (i.e. model updating; BS) in primary somatosensory cortex.**

Abstract:

From around 70ms post-stimulus onset, secondary somatosensory cortices are found to represent confidence-corrected surprise as a measure of model inadequacy. **Indications of Bayesian surprise encoding, reflecting model updating, are found in primary somatosensory cortex from around 140ms.**

Further minor edits to the phrasing of the results can be found at l.592, 634, 638, 644, 701.

Other issues:

- You write in the abstract:

"As such, this dissociation indicates that early surprise signals may control subsequent model update rates."

I don't see how this is indicated by your data/results. It is a plausible interpretation.

We thank the reviewer for pointing this out and now write

Changes in revised manuscript (Abstract):

This dissociation is compatible with the idea that early surprise signals may control subsequent model update rates.

- In your response letter:

"That is to say, the currently tested models do not provide a plausible manner by which the brain acquires the estimated transition probabilities and subsequent surprise quantities. Rather, we view our model comparison as a methodology to infer on qualities that a future successful neural algorithm is likely to exhibit (e.g. using estimated transition probabilities to compute an early puzzlement surprise signal scaled by confidence)."

I appreciate this perspective and think it would be worthwhile sharing this with the reader as well.

We agree with the reviewer that this perspective should be integrated into the manuscript and have added it to our discussion.

Changes in the revised manuscript (l. 760):

The sort of computations (relating to surprise and belief updating) and learning models we consider might be viewed in light of theories such as predictive coding and the free energy principle for which preliminary work suggests implementational plausibility (e.g. [93]). **The computation models tested in the current study do not provide a biophysically plausible manner by which the brain acquires the estimated transition probabilities and subsequent surprise quantities. Rather, the models serve to identify qualities that a future successful biophysically plausible algorithm should exhibit.**

Reviewer #3:

I would like to thank the authors for their explanations and additional analysis, which clearly help understand the results and also support many of their conclusions. However, I would encourage them to include more important information about statistics in the main manuscript (for example the expected posterior probabilities and the protected exceedance probabilities are provided to the supplement). Finally, there still remain some open questions regarding the hierarchical scheme, model comparison and thresholds which I think were not answered sufficiently, yet.

We thank the reviewer for their continued efforts. We aimed to resolve the remaining open questions in the revised manuscript detailed in our responses to both reviewers.

Major:
Hierarchical scheme: First of all, thank you for this extensive reply. I used the term hierarchical in order to reflect how you named it in the original submission it was not my intention to imply that this was a truly hierarchical Bayesian scheme. You have decided to stick with the "hierarchical model selection" scheme. I still think, this is not a standard in DCM research. There might be some papers which have used family model comparison in this way, but to my best knowledge there is no theoretical or methods paper suggesting this. I do not recall the original paper by Penny and colleagues does promote family comparison for this purpose either. If you are aware of one or several citations that advocate such a hierarchical selection approach and evaluate it critically, I would be more than happy to know it, and you should cite them in your paper. Having said this, your approach is reminiscent of using orthogonal contrasts in a factorial design to reduce the search space and if one would assume that model selections are orthogonal (which I think one can) selecting certain time points based on one comparison and then restricting the rest of the analysis to those should be fine. However, the combined reduction of search volume and model space is to my best knowledge a novel approach. Hence, you should discuss it critically.

We acknowledge that the approach we applied to reduce data and model spaces is not standard and the suggestion to discuss it critically is well taken. The step-wise model selection procedure was intended to provide an arbitrary but interpretable way of creating summary statistics of very large data and model spaces. We aimed to emphasize this by changes in the methods and results section of the revised manuscript.

Methods section - Bayesian model selection (l. 448, 458):

On this final level, surprise read-out functions were compared for the winning model class and corresponding inference type. The direct comparison of read-out models within the winning family allows for the use of protected exceedance probabilities (currently not available for family comparisons), which provide a robust alternative to inflated exceedance probabilities [67]. The step-wise procedure allows for spatio-temporal inference on particular read-out functions for which there is evidence

for a belonging model class and inference type, facilitating the interpretation of the results. The hierarchical ordering thus moves from general to specific principles: the model class and inference type determine the probability estimates of the model, which are finally read out through surprise computation. **While this procedure provides a plausible and interpretable approach to our model comparison, it should be noted that it constitutes an arbitrary choice in order to reduce data and model space and must be interpreted with caution. As a supplementary analysis, we performed non-hierarchical (factorial) family comparison analysis (S5 Fig) which groups the entire model space into the respective families for each family comparison without step-wise data reduction.** The same procedures were used for the EEG sensor and source data.

Results section - Modeling in sensor space (l. 625):

In an additional analysis, we performed a non-hierarchical model comparison which grouped the entire model space in the respective families of interest without step-wise data reduction. These results (S5 Fig) broadly replicate the findings from the hierarchical approach across the levels and likewise indicate that the order of transition probability (TP1 and TP2) can not be resolved in early time windows.

Discussion section (l. 902):

Furthermore, some limitations might concern the stepwise model comparison intended to yield interpretable results by allowing inference on the generative model giving rise to surprise signatures. A reduction of both data and model space is not a standard procedure in Bayesian model comparison and we stress that we do not provide a methodological validation of this approach. Nevertheless, we argue that this scheme capitalizes on the hierarchical structure of the model space, provide model recoverability simulations, and present similar results using a standard factorial family comparison to support that the main conclusions are not dependent on the exact model comparison approach.

I have some additional comments to your answers to my requests for clarification on the hierarchical scheme. None of these points is new. They are all related to your answers to the previous comments.

Regarding thresholds you say: "We allowed for lower thresholds for these second and third analysis steps on remaining data given a threshold had already been applied." I do not understand this rationale. Why should a threshold on a lower level be less stringent than on a higher level of your selection? If these are orthogonal questions, which is how you treat them in your comparison, all comparisons should have the same threshold, I would think. It would be much more convincing if you used the same exceedance probability threshold for all levels (for example 0.95, which would roughly correspond to a p-value of 0.05 (see also my comment below)). If you stick to the thresholds you selected, I think you should remove the above sentence and make clear that this was an arbitrary choice.

We thank the reviewer for their comment and agree that results with exceedance probabilities above 0.95 should be clearly demarcated and have correspondingly adjusted thresholding in the revised manuscript. As the main concern seems to be addressing thresholding that is potentially too loose, we decided to stick with a high threshold of EP>0.99 for the first model comparison of [NULL vs DC vs HMM] to be conservative against the null-model. For the subsequent comparisons of [TP1 vs TP2] and [SP vs AP vs TP1] we threshold at 0.95. The final surprise comparison depicted as electrode topographies are provided with demarcations of electrodes that survive a thresholding of protected exceedance probabilities above 0.95. The topographies are otherwise shown without thresholding in order to indicate tendencies of the surprise readouts. As the thresholding

indeed does not serve to indicate statistical significance, we include in the manuscript that the thresholds were an arbitrary choice to draw conclusions on (and visualize) the effects with certain minimum effect sizes. We kindly refer the reviewer to the revised Fig. 10 (and Fig. 11 for source space results) shown in response to reviewer #2 above as well as the corresponding changes in the revised manuscript which we provide here again for convenience.

Changes in the revised manuscript:

Methods (l. 438):

A threshold of exceedance probabilities $\varphi > 0.99$ in favour of either the DC or HMM was applied**, so that only whenever there was strong evidence in favour of one of the model classes over both the alternative and the null-model the following analyses were applied.**

Methods (Bayesian model selection, l. 440):

As the current analyses are not statistical tests per se, the thresholding of the data by certain exceedance probabilities ultimately constituted an arbitrary choice to reduce data in order to visualize (and draw conclusions on) effects with certain minimum effect sizes within a large model space.

Similarly, further references to the thresholds have been adjusted to reflect the new thresholding in lines 492 and 604.

Regarding statistics and thresholding: I think you should mention expected posterior probabilities and protected exceedance probabilities directly in the manuscript. The reader should have an idea of the size of the effect from reading the main text. While there is not a one to one mapping from to exceedance probability or to protected exceedance probabilities, you could say something like: "For the individual levels we thresholded at phi = 0.99 which roughly corresponded to = 0.7??, phi = 0.95 (= ??? etc.), and phi = 0.9 (= ???, protected exceedance probability = ???) and phi = 0.7 (= ???, protected exceedance probability =)." You could then refer to the supplementary figure to illustrate all time points and electrodes. I think it would be best, if you used protected exceedance probabilities for the final level. Why would you apply a measure that we know is inflated if you have a robust alternative? From the figure you show in the supplement it seems that there is not much information in the data to distinguish the surprise models. Rigoux and colleagues suggest that one minus the protected exceedance probability could be used similar to a p-value. Hence, an unprotected exceedance probability threshold of 0.9 is low (this is comparable to a p-value of 0.1, but still not considering the null hypothesis of all models being equally likely) and a threshold of 0.7 (p<0.3) seems extremely low. I think this should be made clear to the reader and it might be more correct to not call these findings significant. I do not think it is critical that there is strong evidence for one particular model, but as it stands I fear you tend to interpret relatively little evidence as a strong finding.

We thank the reviewer for this comment and agree that protected exceedance probabilities and expected posterior probabilities, as the more robust statistics, should be presented in the main manuscript. In the revised manuscript, the results of the surprise read outs in Fig. 10 and Fig. 11 now show the protected exceedance probabilities. Additionally, throughout the manuscript protected exceedance probabilities and expected posterior probabilities are made explicit by providing the corresponding values in the main text. Further, in the revised manuscript, we emphasize that the effects are indications as to which type of surprise read-out of the TP model is more likely to be instantiated at particular time-points only, and not probabilistic guarantees. In that regard, we also chose to refrain from the concept of

statistical significance to prevent the implication of guarantees comparable with classical testing terminology. Particularly, any effects after 200 ms post-stimulus onset are pointed out to be very weak and are correspondingly only mentioned insofar as they align with late surprise effects previously reported in the literature (commonly interpreted as the P300). However, some EPs (in the previous version) and (to a lesser extent) protected EPs are indeed above 0.95. This was not very well conveyed in our earlier manuscript as a threshold of 0.9 (and 0.7 respectively) was chosen for illustration purposes while the colorbar scaling did not resolve the peak probabilities well. We kindly ask the reviewer to refer to the reply to reviewer #2 above for the respective changed figures (Fig. 10, Fig. 11).

Changes in the revised manuscript:

Results section (Modelling in sensor space; l.579, 589, 592):

For large time windows at almost all electrodes there is strong evidence in favor of the DC model class (φ > 0.99), while the HMM model class **does not exceed thresholding anywhere**, therefore excluding HMM models from further analyses. **The corresponding threshold of expected posterior probabilities to arrive at comparable results lies around $\langle r \rangle$>0.75 (see S8 Fig).**

For the DC model, TP1 is found to outperform TP2 **(φ>0.95, roughly corresponding to $\langle r \rangle$ >0.7)**, excluding TP2 for the second and third level analyses. In the following step, TP1 clearly performed better than SP and AP at almost all electrodes and time points (see Fig 10A-C0; **φ>0.95 roughly corresponding to $\langle r \rangle$>0.7)**.

Finally, the fact that more stringent statistics like protected exceedance probability reveal a similar pattern although at lower values should not be taken as a confirmation of the inflated values of the exceedance probability. This is what you seem to suggest: "Despite these statistics being diminished, they yield highly similar conclusions, suggesting the results are not solely due to exceedance probability inflation." Of course, the conclusions depend on thresholds. Overoptimistic values of exceedance probability will not change the overall pattern of the maps, but could potentially make us overoptimistic about conclusions. For example, the protected exceedance probability maps suggest that there does not seem to be much evidence in favor of any of the surprise models.

We thank the reviewer for pointing this out and we now instead report the protected exceedance probabilities in the revised manuscript. While it is true that the protected exceedance probability maps provide a less clear picture of the different surprise read-outs than for the other three model comparisons, particularly the early confidence-corrected surprise effects show high protected EPs above 0.95 at some electrode clusters. Nevertheless, throughout the manuscript we adjusted the confidence in the surprise readout conclusions and clarified that the evidence for the intermediary BS effect is weak and that there is no strong evidence for a particular surprise model after 200ms. For convenience we here again provide the relevant changes to the revised manuscript as shown in response to reviewer #2.

Results section - Modeling in sensor space (l. 592):

Thus, the following section presents the random-effects Bayesian model selection results of the readout functions of the Dirichlet-Categorical TP1 model (shown in Fig 10D). The scalp topographies depict the winning readout functions of the DC TP1 model at different time windows. Given the difference in temporal dynamics of faster, early (<200 ms) and slower, late (e.g. P300) EEG components, different time windows were applied for averaging. Early clusters were identified by averaging protected

exceedance probabilities over 10ms windows and using a minimum cluster size of two electrodes, while 50ms time windows were applied for averaging across later time windows with a minimum cluster size of four. The resulting clusters indicate that from around 70ms on, early surprise effects represented by confidence corrected surprise (CS) best explain the EEG data on contralateral and subsequently ipsilateral electrodes up to around 200ms. As demarcated in the plot, the early CS clusters include electrodes with $\tilde{\varphi}$>0.95, which is indicative of a strong effect size. A weaker cluster of Bayesian surprise (BS) is apparent at centro-posterior electrodes between 140-200ms of which the peak electrodes around 150ms show $\tilde{\varphi}$ between 0.8 and 0.95. As such, the mid-latency BS effect is less strong than the earlier CS clusters and can only provide indications. At the time windows of the P300 around 300 and 350ms, similar centro-posterior electrodes represent weak Bayesian surprise (peak $\tilde{\varphi}$ around 0.75) and predictive surprise (PS) clusters (peak $\tilde{\varphi}$ around 0.72), respectively. The mid-latency BS cluster is temporally in accordance with the putative N140 MMR while the late two clusters of BS and PS might be interpreted as indicative of a P300 MMR. However, especially the weak late clusters do not provide clear evidence in favour of a specific surprise readout function.

Discussion:

l. 662:

Using computational modelling, EEG signals were best described by a non-hierarchical Bayesian learner performing transition probability inference. **Furthermore, we provide evidence for an early representation of confidence-corrected surprise localized to bilateral S2 and weak indications for subsequent Bayesian surprise encoding in S1.** These computations were shown to use a local, rather than global, timescale of integration.

l. 821:

The BS effect around 140ms was less pronounced in source space only peaking at $\tilde{\varphi}$ of 0.89 and was localized to S1. Despite the weak evidence for this BS representation around a 140ms somatosensory MMR, its timing matches prior work using Bayesian modeling of surprise signals in the somatosensory system [13].

l. 929:

In conclusion, we **show** that signals of early somatosensory processing can be accounted for by (surprise) signatures of Bayesian perceptual learning. The system appears to capture a changing environment using a static latent state model that integrates evidence on a local, rather than global, timescale and estimates transition probabilities of observations using first order dependencies. **In turn, we provide evidence that the estimated statistics are used to compute a variety of surprise signatures in response to new observations, including both puzzlement surprise scaled by confidence (CS) in secondary somatosensory cortex and weak indications for enlightenment surprise (i.e. model updating; BS) in primary somatosensory cortex.**

Abstract:

From around 70ms post-stimulus onset, secondary somatosensory cortices are found to represent confidence-corrected surprise as a measure of model inadequacy. **Indications of Bayesian surprise encoding, reflecting model updating, are found in primary somatosensory cortex from around 140ms.**

Further minor edits to the phrasing of the results can be found at l.592, 634, 638, 644, 701.

Finally, I think the family comparison where you stick to the factorial design is indeed convincing. And supports your findings within the DC group: TP1 is the clear winning family. Again, as in the hierarchical selection procedure, there seems to be rather little evidence in favor of any particular surprise model. I have one small question for clarification. Did you really not reduce data (the time points and electrodes) in this comparison, or did you just not reduce the model space?

We thank the reviewer for requesting clarification. Indeed, we did not reduce data in the non-hierarchical analysis where all family comparisons concerned both the full model space and all of the data (without reduction of time points or electrodes).

From all the points raised above, I would conclude the following. You can provide robust evidence that the DC is favored over NULL and HMM. In addition, it is still quite clear that TP1 outperforms (TP2, AP and SP) models. For the different surprise models, the evidence seems to get rather weak. One could maybe talk about a tendency of some models to win.

We thank the reviewer for their suggestions. We generally agree and conclude in similar spirit to provide evidence for a static state model (DC) estimating first order transition probabilities (TP1). While it is correct that differences between the surprise readouts become less well pronounced, we do report evidence for early CS over the other surprise models. We intended to make this clear through the aforementioned adjustments to the reported confidence in the results.

Multiple comparison: I think this needs more discussion. I am not aware of work that says that performing thousands of independent Bayesian analyses cannot result in an issue of multiple tests. I am more than happy to be corrected on this. I interpret the statement in the Friston 2002 paper you cite differently. Their setting differs in two important aspects from your analysis. First, it is about parameter estimates. Second, and more importantly, the comment about solving the multiple comparison problem is made in the context of a hierarchical model (PEB) where the higher level serves to set the prior according to the distribution over all other voxels (empirical Bayes). It is this step that "provides" the correction. I do not think the situation is the same in your setting. I think one needs to acknowledge the fact that this is an unsolved problem and discuss it accordingly.

We thank the reviewer for their comment. A similar concern was raised by reviewer #2 and we acknowledge that we do not provide a corrective measure for the large amount of independent model comparisons that are performed. However, these model comparisons do not constitute statistical tests per se, as they do not provide a mapping from the data to binary outcomes. Rather, the resulting exceedance probabilities are a measure of effect size, which are reported here only above a given threshold. It follows that the analyses do not suffer from a classical multiple testing problem, which can be addressed using the control of multiple testing error rates (e.g. the control of the family-wise error rate for fMRI inference based on random field theory). We regretfully referred to statistical significance in the discussion of the previous manuscript, which we avoid in the revised version. Nevertheless, we think it would be very valuable for methodological advances to consider the possibility of randomly occurring high effect sizes given a large number of independent model comparisons. As this is an unsolved problem and currently not methodologically accounted for we conclude to present here only preliminary evidence and make this now explicit in the revised manuscript. For convenience we include here the relevant change as initially presented above to reviewer #2.

Changes in the revised manuscript (Discussion, l. 910):

The analyses performed here include a large number of independent Bayesian model comparisons (as is not uncommon in neuroimaging), yet no corrections are applied. These model comparisons do not constitute statistical tests per se, as they do not provide a mapping from the data to binary outcomes. Rather, the resulting exceedance probabilities are a measure of effect size, which are reported here only above a given threshold. It follows that the analyses do not suffer from classical multiple testing problem, which can be addressed using the control of multiple testing error rates (e.g. the control of the family-wise error rate for fMRI inference based on random field theory). Nevertheless, it would be valuable for methodological advances to consider the possibility of randomly occurring high effect sizes given a large number of independent model comparisons. A multilevel scheme which adjusts priors over models, rather than the current ubiquitous use of flat priors, may be developed as a satisfactory approach (Friston et al., 2002; Gelman et al., 2012; Neath, et al., 2018). As the current method is agnostic to the large number of model comparisons we need to stress that we only report preliminary evidence.

Minor:
Fitting of tau: If there is a citation where your heuristic to correct for the fitting of tau is suggested, you should cite it here. Otherwise, please state that it is a heuristic that somehow punishes for the additional fitting but that one would have to do include tau as an additional parameter in the model fitting to do proper model comparison including tau.

We appreciate the concern and have made it explicit that our tau-penalization is a heuristic approach.

Changes in the revised manuscript (l. 426):

To allow for the possibility that the brain estimates statistics computed across multiple timescales of integration [9, 63, 64], the forgetting-parameter τ of the DC model was optimized for each subject, model, and peri-stimulus time-bin. To this end, DC model regressors were fitted for a logarithmically spaced vector of 101 τ-values on the interval of 0 to 1 and the value of τ that resulted in the highest model evidence was chosen. To penalize the DC model for having one of its parameters optimized, the degree to which τ optimization on average inflated model evidences was subtracted prior to the BMS procedure. Specifically, the difference in model evidence between its average for all parameter-values and the optimized value was computed and subsequently averaged across post-stimulus timebins, sensors, and subjects. **It should be noted that the applied procedure constitutes a heuristic for the penalization of model complexity while no explicit parameter fitting procedure was implemented within model estimation.**

In supplementary figure 4, it looks as if a threshold of 0.9 was used to go from level 2 to level 3. However, in the manuscript and the corresponding figure you write 0.95. Please correct the one that is wrong.

We thank the reviewer for pointing out the typo in figure S4 Fig (now S5 Fig.) and have corrected it accordingly.

The simulation you perform is illustrative but difficult to assess without knowing more details. In particular, it would be interesting to know how you simulated different settings of families. Did you sample from a Dirichlet distribution using the posterior of your analysis or did you assume that all 40 subjects have the same model. The latter would probably be quite an extreme case. In any case, I am not so sure a simulation can prove that the method is

correct. But, it can already show some limitations, which seem to occur for certain models even in this ideal scenario.

We thank the reviewer for pointing out the lacking information in this section. The simulation indeed assumes that all subjects use the same model, which we now make explicit in its description. Given the limited cognition involved in the distractor task and the task-irrelevancy of most of the stimuli, we consider a limited role for cognitive strategies that may otherwise vary across subjects. Thus, we did not expect large computational variability between subjects in the relatively early EEG signatures focused on here, which motivated this simpler approach. The choice for random-effects BMS was largely driven by its advantage in protecting against outliers, rather than by a belief of interindividual differences in the used models between subjects. Nevertheless, the reader should indeed be informed of this assumption (and potential limitation).

<u>Changes in the revised manuscript (l. 492, 499):</u>

For each noise level, we generated 40 data sets (corresponding to the number of subjects) to apply our random-effects model comparison analyses. This process was repeated 100 times for each of the different comparisons: null model vs DC model vs HMM (C1), DC TP1 vs TP2 (C2), DC SP vs AP vs TP1 (C3), and DC TP1 PS vs BS vs CS (C4). Family and model retrieval using exceedance probabilities worked well across all levels (S6 Fig), with a bias to the null model as signal-to-noise decreases. By inspecting the posterior expected values of B_2 and \lambda^{-1} which resulted from fitting the model regressors to the EEG data, an estimate of the signal-to-noise ratio that is representative of the experimental work can be obtained. By applying the thresholds of φ > 0.99, φ > 0.95, φ > 0.95, and $\tilde{\varphi}$ **> 0.95** across the four comparisons respectively and subsequently inspecting the winning families and models at 2 = 750 (i.e., an SNR of 1/750), no false positives were observed. For C1 and C4, recovery was successful for all true, but unknown models in all of the 100 instances. While for C2 and to a lesser extent C3, concerning the families of estimated sequence statistics, false negatives were observed only when confidence-corrected surprise was used to generate data. For C2, this led to false negatives in 67 (TP1 CS) and 55 (TP2 CS) percent of cases, while for C3 28 (SP CS), 0 (AP CS), and 33 (TP1 CS) percent false negatives were observed. **Each set of 40 data sets was generated with the same true, but unknown model. Due to the limited cognitive flexibility afforded by the distractor task, we did not expect large variability in the models used across subjects. Nevertheless, if this assumption is incorrect these simulations potentially overestimate the recoverability of the different models.**

In summary, I still consider this a highly valuable contribution for PLOS CB, but I would think that the issues raised above should be considered.

At this point we would like to thank the reviewers once again for their enormous help, which is rarely found in revisions in such a detailed and constructive form.
